# 3D-Printing of Magnetoactive Gelatin–Alginate Scaffolds

**DOI:** 10.3390/pharmaceutics18091186

**Published:** 2026-09-19

**Authors:** Sofía Municoy, Exequiel Giorgi, María Edith Farías, Romina B. Currá, Hina N. Chaudhari, Rajshree B. Jotania, Robert C. Pullar, Mauricio De Marzi, Martín F. Desimone

**Affiliations:** 1 Universidad de Buenos Aires, Facultad de Farmacia y Bioquímica, Instituto de Química y Metabolismo del Fármaco (IQUIMEFA), Consejo Nacional de Investigaciones Científicas y Técnicas (CONICET), Junín 956, Buenos Aires 1113, Argentina; smunicoy@docente.ffyb.uba.ar (S.M.); desimone@ffyb.uba.ar (M.F.D.); 2 Grupo de Investigaciones Básicas y Aplicadas en Inmunología y Bioactivos (GIBAIB), Instituto de Ecología y Desarrollo Sustentable (INEDES), CONICET, Universidad Nacional de Luján, Av. Constitución y Ruta 5, Luján 6700, Buenos Aires, Argentina; egiorgi@unlu.edu.ar; 3 Laboratorio de Inmunología, Departamento de Ciencias Básicas, Universidad Nacional de Lujan, Av. Constitución y Ruta 5, Luján 6700, Buenos Aires, Argentina; 4 Grupo de Investigación en Nanopartículas y Reología de Biopolímeros Alimenticios (GINaRBA), Departamento de Tecnología, Universidad Nacional de Luján, Av. Constitución y Ruta 5, Luján 6700, Buenos Aires, Argentina; efarias@unlu.edu.ar (M.E.F.); rcurra@mail.unlu.edu.ar (R.B.C.); 5 Department of Physics, University School of Sciences, Gujarat University, Ahmedabad 380009, India; heenachaudhary0506@gmail.com (H.N.C.); rajshree_jotania@yahoo.co.in (R.B.J.); 6 Department of Molecular Science and Nanosystems (DSMN), Università Ca’ Foscari, Venezia, 30172 Venice, Italy; robertcarlyle.pullar@unive.it

**Keywords:** U-type hexaferrite particles, magnetic particles, gelatin, alginate, 3D printing

## Abstract

**Background/Objectives:** Magnetically responsive biomaterials have emerged as promising platforms for tissue engineering as they allow remote stimulation of cells and improved control over tissue regeneration. Although gelatin–alginate hydrogels incorporating iron oxide nanoparticles have been reported, the use of U-type hexaferrite particles, particularly Al^3+^-substituted compositions, remains largely unexplored. This study aimed to develop and characterize extrusion-based 3D-printed gelatin–alginate scaffolds containing U-type hexaferrite particles and to evaluate the influence of particle composition and loading on the rheological, physicochemical and magnetic properties of the resulting biomaterials, as well as their in vitro compatibility with macrophages. **Methods:** Gelatin–alginate inks containing two U-type hexaferrite compositions (Ba_4_Co_2_Fe_36_−*_x_*Al*_x_*O_60_; *x* = 0.0 and *x* = 1.0) at two particle loadings (20 and 200 mg) were prepared and processed by extrusion-based 3D printing. The scaffolds were characterized by rheological analysis, SEM-EDS, FTIR, swelling measurements, magnetic responsiveness, and in vitro biological evaluation using RAW264.7 macrophages. **Results:** The inks exhibited suitable shear-thinning behavior and viscoelastic properties for extrusion-based printing. Increasing particle loading enhanced the thermal resistance of the network, whereas Al^3+^ substitution modified the viscoelastic response of the polymeric network. The 3D scaffolds successfully responded to an external magnetic field and SEM-EDS confirmed the homogeneous incorporation of hexaferrite particles. The magnetic scaffolds did not compromise macrophage metabolic activity. **Conclusions:** U-type hexaferrite particles provide an effective strategy for producing 3D-printed magnetically responsive scaffolds with tunable rheological properties without inducing an inflammatory response. The combined modulation of particle composition and loading represents a versatile approach for designing multifunctional inks with potential applications in tissue engineering.

## 1. Introduction

The development of tissue engineering has progressively shifted from the fabrication of passive structural substitutes to the design of multifunctional biomaterials capable of actively regulating the cellular microenvironment. Within this context, magnetic biomaterials (MBMs) have emerged as a promising platform, as they allow the application of remotely controlled physical stimuli to modulate cellular responses and enhance tissue regeneration with high spatial and temporal precision [1]. In this sense, MBMs represent a novel and versatile opportunity in regenerative medicine due to their ability to respond to externally applied magnetic fields. This distinctive feature provides opportunities for the remote modulation of therapeutic agent delivery, regulation of mechanobiological signals, and the generation of localized thermal effects upon demand [2,3,4].

MBMs combine magnetic elements, usually composed of iron (Fe), nickel (Ni), and cobalt (Co), with biocompatible matrices such as polymers, hydrogels, or 3D-scaffolds [5]. Among Fe-based magnetic materials, hexaferrites are of particular interest because of their unique combination of structural and magnetic properties [6,7]. Magnetite (Fe_3_O_4_) and maghemite (γ-Fe_2_O_3_) are cubic spinel ferrites with low magnetocrystalline anisotropy, and are usually used as superparamagnetic nanopaticles with no residual magnetization without an applied field (zero coercivity). Hexaferrites, such as U-type ferrites, have a uniquely high magnetocrystalline anisotropy, producing much stronger magnetic anisotropy fields than the cubic magnetite/maghemite, and giving large coercivity values. This prevents spontaneous demagnetization in the absence of an applied magnetic field, and allows for highly efficient mechanical rotation under external magnetic triggers [1]. Hexaferrites can also have enhanced heat generation for specialized magnetic hyperthermia treatments, and whereas magnetite can struggle to generate sufficient heat in low-concentration or extracorporeal environments, the high coercivity of hard hexaferrites translates to larger magnetic hysteresis loops, maximizing specific loss power (SLP) for localized tumor removal [8,9]. Unlike conventional magnetite, hexaferrites possess a complex hexagonal crystal structure whose magnetic and dielectric properties can be readily tuned through ionic substitution, while maintaining a controlled particle morphology. The substitution of aluminum into the hexaferrite structure is known to be able to promote unixial magnetocrystalline anisotropy and increase coercivity, and thus the aspects above for biomedical applications.

A wide variety of biomaterials are used for MBMs, including naturally derived polymers. Collagen [10], gelatin [11], alginate [12] and chitosan [13,14] have gained considerable attention. Due to their inherent biocompatibility, biodegradability, low cost, bioavailability and structural similarity to the native extracellular matrix, they provide a favorable microenvironment for cell adhesion, proliferation, and differentiation while offering tunable physicochemical and mechanical properties that can be adapted to the requirements of different regenerative applications [15,16,17]. Alginate (Alg) is a hydrophilic and anionic polysaccharide extracted from brown algae. Its molecular structure is composed of linear chains of β-D-mannuronic acid (M) and α-L-guluronic acid (G) residues connected through 1→4 glycosidic linkages, which are responsible for its hydrogel-forming ability in the presence of divalent cations (Ca^2+^, Mg^2+^, etc.) [18]. Alg-based hydrogels possess high swelling capacity and flexibility which make them suitable matrices for supporting cell growth and promoting vascular tissue development. Gelatin (Gel) originates from collagen, the principal protein of the extracellular matrix [19]. It has been employed in a wide range of tissues as it favors cell adhesion, differentiation and proliferation while it is enzymatically degraded by metalloproteinases without eliciting an adverse immune response. The combination of Alg and Gel has been extensively explored for the development of MBMs for tissue engineering, as Alg can contribute to the formation of a porous network that may facilitate the diffusion of nutrients and bioactive molecules, whereas gelatin promotes cell adhesion and supports cell proliferation and differentiation [20,21,22].

Tissue engineering is being transformed not only by the development of these advanced bioactive materials with response to an external stimulus, but also by the emergence of innovative fabrication technologies that enable the generation of complex architectures with improved structural and biological properties. Among these technologies, 3D printing has emerged as a powerful tool for the precise design and manufacturing of biomaterial-based scaffolds, offering unprecedented control over their geometry, composition, and functionality [23,24]. The synergy between MBMs with the advanced 3D printing approach has further expanded the capabilities of tissue engineering by enabling the fabrication of four-dimensional (4D) magneto-responsive constructs that can be remotely activated by external magnetic fields [25,26,27]. Compared with conventional scaffolds, these magnetic platforms exhibit tunable rheological properties, improved mechanical performance and provide dynamic physical cues that enhance cell adhesion, proliferation, and differentiation [28]. Such systems offer a non-contact strategy for mechanical stimulation, allowing precise modulation of the cellular microenvironment and, consequently, cell behavior [29,30].

Given the growing interest in developing biomaterials with advanced functionalities to promote tissue regeneration, the combination of 3D printing with bioactive materials has emerged as a highly attractive strategy. Although magneto-responsive Gel-Alg hydrogels incorporating iron oxide nanoparticles have been widely investigated, studies combining hexaferrite magnetic particles (HMP) with 3D-printed Gel-Alg matrices remain limited. Moreover, to the best of our knowledge, the use of Al^3+^-substituted U-type hexaferrites in 3D-printed biomaterial applications has not been previously reported. While magnetite (Fe_3_O_4_) and maghemite (γ-Fe_2_O_3_) are widely explored in biomedical applications, U-type hexagonal ferrites (Ba_4_Co_2_Fe_36_−*_x_*Al*_x_*O_60_) offer distinct advantages for structural tissue engineering scaffolds. Unlike iron oxides, hexaferrites are highly stable and resistant to oxidation/corrosion, whereas magnetite can degrade to other phases in physiological fluids [8,9,31]. Furthermore, hexaferrites can absorb high-frequency microwave (GHz) wavelengths, offering the potential for dual-mode diagnostics and simultaneous therapy options (theranostics), for example when combined with magnetic resonance imaging (MRI) targeting, which magnetite or other spinel ferrites alone cannot. The inclusion of aluminum substitution (*x* = 1.0 compared to the unsubstituted *x* = 0.0 composition) serves to fine-tune the magnetic properties of the ceramic phase. The superior magnetic anisotropy, chemical stability, and tunable magnetic properties of U-type hexaferrites make these materials attractive candidates for the development of next-generation magnetically responsive scaffolds, providing a basis for future studies on magnetically assisted modulation of scaffold properties and cellular responses. To investigate the influence of the magnetic phase on both the physicochemical properties of the Gel-Alg-based inks and in vitro cellular compatibility of the resulting 3D scaffolds, two U-type hexaferrite compositions, without (*x* = 0.0) and with (*x* = 1.0) Al^3+^ substitution levels, were selected. In addition, each composition was incorporated at two different particle loadings (20 and 200 mg), allowing a systematic evaluation of the effects of both ferrite composition and concentration on the rheological behavior of the inks and magnetic properties of the 3D-printed scaffolds. As an initial assessment of their cellular compatibility, macrophage metabolic activity was evaluated after exposure to the 3D-printed scaffolds. Further studies will be required to determine whether their magnetic responsiveness can be exploited to actively modulate cellular behavior through controlled application of external magnetic fields.

## 2. Materials and Methods

### 2.1. Magnetic Particles

#### 2.1.1. Preparation

U-type hexaferrite powders with the chemical composition Ba_4_Co_2_Fe_36_−*_x_*Al*_x_*O_60_ (*x* = 0.0, and 1.0) were synthesized by the citrate auto-combustion method [6]. Aluminum nitrate (Al(NO_3_)_3_·H_2_O, 99.9%, Sigma-Aldrich, St. Louis, MO, USA), cobalt nitrate (Co(NO_3_)_2_·6H_2_O, 99.99%, Merck, Darmstadt, Germany), barium nitrate (Ba(NO_3_)_2_, 99.0%, Loba Chemie, Mumbai, India), ferric nitrate (Fe(NO_3_)_3_·9H_2_O, 99%, HPLC), and citric acid (C_6_H_8_O_7_·H_2_O, HPLC grade, 99%) were used as starting reagents. The molar ratio between the total metal nitrates and citric acid was maintained at 1:1. Briefly, the required stoichiometric amounts of the metal nitrates were individually dissolved in double-distilled water and subsequently combined under continuous magnetic stirring. A citric acid solution was then added dropwise to the mixed nitrate solution, followed by adjustment of the pH to 7 using a 25% (*w*/*v*) aqueous ammonia solution, promoting the formation of metal–citrate complexes. The resulting solution was heated at 100 °C until a viscous brown gel was obtained. Upon further heating, the gel underwent spontaneous auto-combustion, yielding a dark brown porous ash.

The combustion product was ground to a fine powder using an agate mortar and pestle, calcined in air at 550 °C for 4 h, and allowed to cool naturally to room temperature. Finally, the powder was heated at 1300 °C for 5 h in air to obtain U-type hexaferrite.

#### 2.1.2. Characterization

The crystalline structure and phase composition of the synthesized powders were investigated at room temperature by X-ray diffraction (XRD) using a SmartLab SE diffractometer equipped with Cu Kα radiation (λ = 1.5406 Å). Diffraction patterns were collected over a 2θ range of 20–80° using a step size of 0.02°. Phase identification and refinement of the lattice parameters were performed with WinPLOT software (version 2006) . The average crystallite size (*D_XRD_*) was estimated from the most intense diffraction peak using the Scherrer equation:

(1) $$D_{XRD}=\;\frac{0.9\;.\;\lambda }{\beta_{1/2}cos\theta }$$

where *D_XRD_* = mean size of the ordered crystalline domains; λ = wavelength of the X-ray source (Cu Kα ≈ 0.15406 nm); *β*_1/2_ = full width at half maximum (FWHM), measured in radians; *θ =* Bragg angle (half of the 2θ peak position).

The morphology of the ferrite powders was examined by field-emission scanning electron microscopy (FE-SEM) using a JEOL JSM-7600F microscope (JEOL Ltd., Akishima, Tokio, Japan). Elemental composition was determined by energy-dispersive X-ray spectroscopy (EDS) using the detector coupled to the same instrument.

Magnetic measurements were carried out at room temperature using a vibrating sample magnetometer (VSM, Lake Shore 7400 Series, Westerville, OH, USA) under an applied magnetic field ranging from −15 kOe to 15 kOe (−1.5 T to +1.5 T). The magnetic parameters, including saturation magnetization (M_s_) and coercivity (H_c_), were obtained from the hysteresis loops, and the magnetic anisotropy constant (K) was subsequently estimated from these values.

### 2.2. Three-Dimensional Printing

#### 2.2.1. Preparation of Gelatin–Alginate Ink

Type B gelatin from bovine skin (Sigma Aldrich, USA) and sodium alginate with the quality and purity standards set by the *Farmacopea Ufficiale* (Carlo Erba reagents S.A.S., Barcelona, Spain) were used. To prepare sterile inks, 0.5 g of each powder was exposed to ultraviolet (UV) light for 30 min. The sterilized powders were then aseptically mixed with 10 mL of sterile water at 37 °C under vigorous stirring. The resulting ink was transferred into a 5 mL syringe and allowed to hydrate overnight at 4 °C prior to use. For the magnetic inks, the biopolymer powders were first mixed with either 20 or 200 mg of the HMP prior to hydration with sterile water. The powder mixtures were then hydrated following the same procedure described above.

#### 2.2.2. Rheological Properties of the Inks

Rheological tests of the magnetic ink were performed using a rheometer (MCR 301, Anton Paar, Graz, Austria). The measuring system was equipped with a parallel-plate geometry (PP25, 25 mm diameter) with a 0.75 mm measuring gap.

Frequency sweep tests were conducted from 0.1 to 10 Hz at a constant strain of 0.1% to determine the storage modulus (G′) and loss modulus (G″) as a function of frequency under oscillatory shear at 20 °C.

The temperature dependence of the ink systems was investigated by monitoring G′ and G″ as a function of temperature at a constant strain of 0.1%, within the linear viscoelastic region (temperature range: 20–36 °C) following the methodology described by Barth et al. [32].

Shear stress and apparent viscosity (η) were recorded as a function of shear rate using a logarithmic shear-rate ramp from 0.1 to 100 s^−1^. All experiments were performed in duplicate at 35.5 °C. To minimize water evaporation during the measurements, the exposed edge of the sample was surrounded with cotton soaked in ultrapure water. The experimental data were fitted to the Power-Law model [33]:

(2) $$\tau =K{\dot{\gamma }}^{n}$$

where τ = shear stress (Pa); $\dot{\gamma }$ = shear rate (s^−1^); *K* = consistency index (Pa·s^n^); n = flow index (adimensional).

#### 2.2.3. Printing Parameters for Magnetic Inks

A square-grid scaffold (15 × 15 × 2 mm) was selected as the model structure to evaluate the properties of the 3D-printed magnetic biomaterials. The printing configuration comprised three layers, including one solid top layer and one solid bottom layer, with a layer height of 0.7 mm and a minimum top and bottom thickness of 0.7 mm. A single external perimeter and 0% internal infill were used. The scaffold was designed using PrusaSlicer^®^ 2.9.1 software and exported as a G-code file, which was used to define the printing path and scaffold geometry. The constructs were fabricated using an extrusion-based Life SI 3D bioprinter (Life SI, Humanizing Technology, Córdoba, Argentina) equipped with a 21 G needle (514 μm inner diameter). Based on preliminary optimization experiments, the printing parameters were set at a printing temperature of 30 °C, a print delay of 150 ms, a material amount (MAT) of 1 nL per 4 μm of travel and the print bed was set to the “high cold” condition (10 °C). The printing speed was controlled through the print delay parameter.

#### 2.2.4. Gelification of 3D-Printed Magnetic Scaffolds

Following 3D printing, the magnetic scaffolds were crosslinked by immersion in a 100 mM CaCl_2_ (ITW Reagents, 99–105%, Barcelona, Spain) solution for at least 15 min. The crosslinked hydrogels were then washed three times with deionized water to remove residual calcium chloride and stored at 4 °C until further use.

#### 2.2.5. Printability Test Methodology

To evaluate the quantitative printability (Pr) of the inks, the following formula was used:

(3) $$Pr=\frac{L^{2}}{16\;A}$$

where L is the perimeter and A is the area. When Pr = 1.0, the ink is in an optimal gelation state, and a continuous, uniform, and smooth extrusion filament is observed, enabling the production of lattices with the desired structure. A value below 1 (Pr < 1) shows under-gelation and spreading, while above 1 (Pr > 1) indicates over-gelation and breaking [34]. To calculate the Pr value of the magnetic inks, top-view images of the 3D-printed scaffolds were captured and the perimeter and area of the grid holes were calculated using ImageJ 1.52f software (n = 10). For each of the ink formulations, an average Pr value and standard deviation (SD) were calculated.

### 2.3. Characterization of 3D-Printed Magnetic Scaffolds

#### 2.3.1. FE- SEM Images and EDS Analysis

The surface morphology and elemental composition of the 3D-printed magnetic hydrogels were characterized by scanning electron microscopy (FE-SEM) using a Carl Zeiss EVO 10 HV W microscope (Carl Zeiss Microscopy GmbH, Jena, Germany) equipped with an Oxford Instruments Ultimax 40 energy-dispersive X-ray spectroscopy (EDS) detector (Oxford Instruments NanoAnalysis, High Wycombe, United Kingdom. Briefly, the scaffolds were fixed in a 2.5% glutaraldehyde solution and freeze-dried for 24 h. The lyophilized samples were mounted on aluminum SEM stubs using double-sided carbon adhesive tape and sputter-coated with gold prior to imaging.

#### 2.3.2. FTIR Analysis Methodology

Fourier transform infrared (FTIR) spectra of the 3D-printed scaffolds were acquired using an FTIR-Raman Nicolet iS50 spectrometer (Thermo Scientific, Waltham, MA, USA). Prior to analysis, the scaffolds were freeze-dried for 24 h, and a small slice of each sample was placed on the attenuated total reflectance (ATR) accessory. The spectra were collected at room temperature over the wavenumber range of 4000–400 cm^−1^.

#### 2.3.3. Swelling Capacity Methodology

The swelling capacity of the 3D-printed Gel-Alg-based hydrogels was evaluated by a gravimetric method. Briefly, the freeze-dried scaffolds were weighed and subsequently rehydrated with distilled water at room temperature. The samples were weighed over a period of 2 h. Before each measurement, the scaffolds were gently removed from the solution and the excess surface water was carefully removed with filter paper. The swelling ratio was calculated using Equation (4):

(4) %Swelling = [(W_wet_ − W_dried_)/W_dry_] × 100

where W_wet_ and W_dry_ correspond to the weights of the hydrated and dried scaffolds, respectively.

### 2.4. Functional Characterization of 3D-Printed Magnetic Scaffolds

#### 2.4.1. Magnetic Response Methodology

The magnetic response of the 3D-printed HMP-containing scaffolds was evaluated by exposing the samples to an external magnetic field of 0.60 Tesla generated by NdFeB permanent magnets. The behavior of the scaffolds in response to the applied magnetic field was visually monitored and recorded.

#### 2.4.2. Immune Cells Response Methodology

##### In Vitro Assays

Macrophage cell line RAW 264.7 (TIB-71™) from American Type Culture Collection (ATCC) were cultured in complete DMEM medium (10% FBS) at 37 °C and 5% CO_2_ atmosphere. To prevent contamination with bacterial endotoxins (such as lipopolysaccharide, LPS), which can artificially activate macrophages, all cell culture plasticware, pipettes, and media components were sterile and certified pyrogen-free. Magnetic scaffolds were handled under sterile laminar flow hoods. The absence of LPS was assessed by Limulus test. For assays, cells were placed in 24-well plates in DMEM medium (10% FBS) at a final concentration of 3 × 10^5^ cells per mL with 1 mL as the final volume. Metabolic cell activity in the presence of 3D-printed scaffolds was measured after 24 h.

##### Measurement of Cellular Metabolic Activity

Metabolic activity was determined by MTT assay in presence of the 3D-printed scaffolds. Briefly, supernatants of cell cultures were extracted and stored, 100 µL of MTT reagent (5 mg/mL) and 400 µL of DMEM were added to the 3D-printed scaffolds and the plates were incubated at 37 °C and 5% CO_2_ for 4 h. After 2 washes with phosphate buffer, absolute ethanol was added to dissolve formazan crystals, and the absorbance of the resultant solution was determined at 570 nm. Results were expressed as mean ± standard deviation for triplicate biological experiments.

##### Nitric Oxide (NO) Expression

When NO is secreted by macrophages, it is quickly oxidized to nitrites and nitrates by oxygen. NO production is proportional to nitrites concentration. Nitrite determination was performed by Griess reaction as described by De Marzi et al. [35].

##### Statistical Analysis

All quantitative data are expressed as the mean ± standard deviation (SD) of at least n = 3 independent replicates. Statistical significance between the control and treated groups at each time point was determined using a one-way analysis of variance (ANOVA) followed by Turkey post-hoc test for multiple comparisons. Differences were considered statistically significant when *p* < 0.05. Specific significance thresholds are reported as * *p* < 0.05, ** *p* < 0.01, and *** *p* < 0.001.

## 3. Results and Discussion

### 3.1. Preparation and Characterization of Hexaferrites Magnetic Particles

The U-type hexaferrite particles employed in this study were previously synthesized and structurally characterized by our research group [6]. Notably, these particles include an Al^3+^-substituted U-type hexaferrite composition, a modification that, to the best of our knowledge, had not been previously reported. This unique composition provides an opportunity to investigate how Al^3+^ substitution influences not only the intrinsic magnetic properties of the ferrite but also the rheological behavior of Gel-Alg inks and the physicochemical and biological performance of the resulting 3D-printed scaffolds.

**Figure 1:**
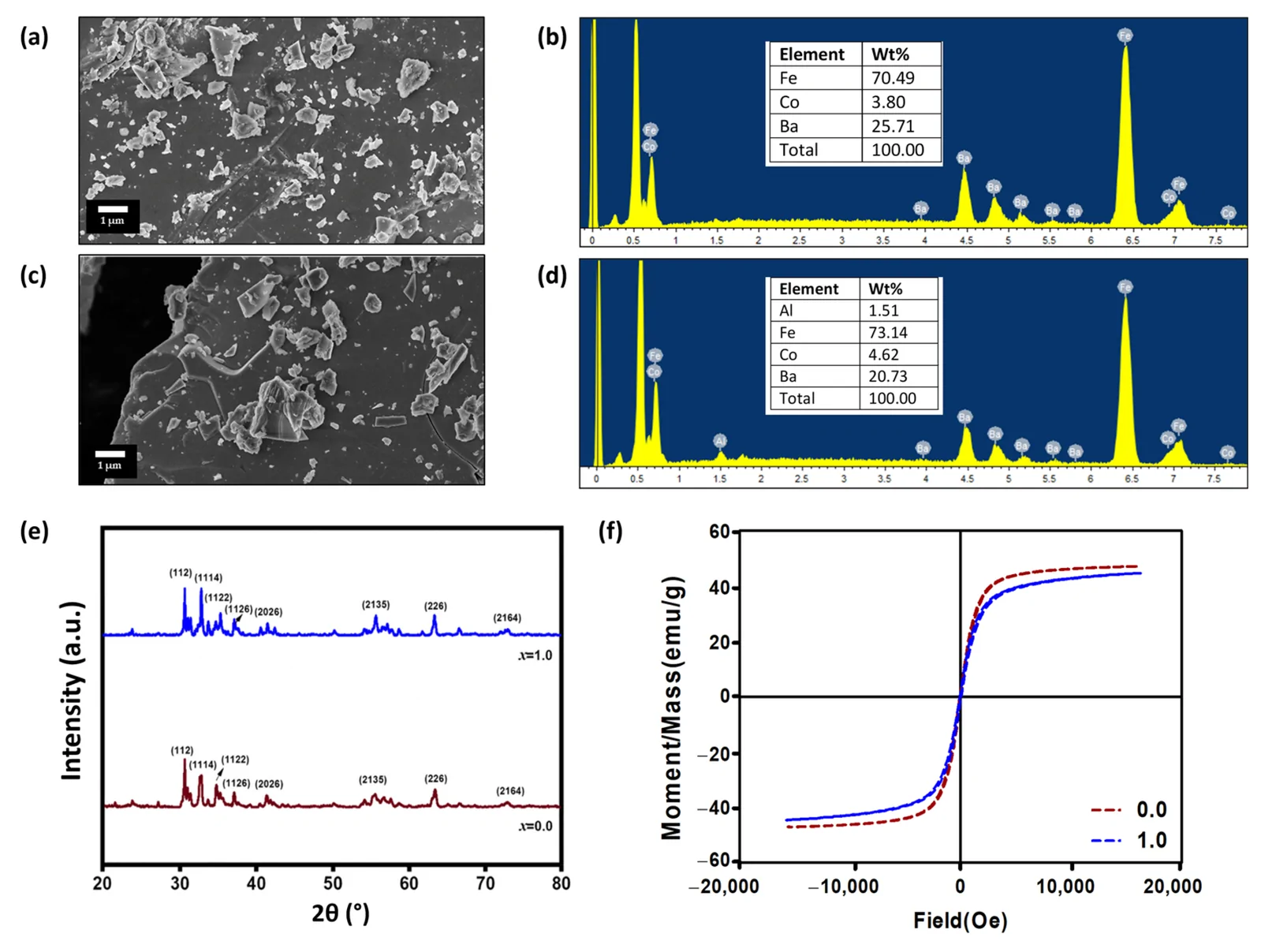
Characterization of the U-type hexaferrite particles. FE-SEM images of the non-substituted (*x* = 0.0) and Al^3+^-substituted (*x* = 1.0) U-type hexaferrites (**a**,**c**), together with their corresponding EDS spectra (**b**,**d**), repectively. (**e**) XRD patterns showing the characteristic diffraction peaks of the U-type hexaferrite structure for both compositions. The arrows indicate the crystallographic planes (hkl) associated with the corresponding diffraction peaks. (**f**) Magnetic hysteresis loops of Ba_4_Co_2_Fe_36−x_Al_x_O_60_ (*x* = 0.0, 1.0) hexaferrite powder samples. Scale bar of (**a**,**c**) reperesents 1 μm. Figures (**b**,**d**–**f**) are addapted from [6], following the term and conditions of the Licenses granted by Copyright Clearance Center, Inc. (“CCC”), Danvers, MA, USA. (https://doi.org/10.1039/d4tc01659a).

U-type hexaferrite particles were prepared by using a citrate-gel auto-combustion technique. The morphology of the selected U-type hexaferrite particles (Ba_4_Co_2_Fe_36_O_60_, *x* = 0.0, and Ba_4_Co_2_Fe_35_AlO_60_, *x* = 1.0) was characterized by FE-SEM and EDS. FE-SEM micrographs (Figure 1a,c) revealed that both compositions consisted of irregular, non-uniform agglomerates with particle clusters in the micrometer range (from 0.3 to 3 μm). These agglomerates are composed of smaller crystallites, indicating the formation of multidomain particles. EDS (Figure 1b,d) confirmed the presence of the expected constituent elements in both samples. The non-substituted ferrite (*x* = 0.0) exhibited characteristic signals corresponding to Ba, Co, Fe, and O (Figure 1b), whereas the Al^3+^-substituted composition (*x* = 1.0) additionally displayed the characteristic Al signal, confirming the successful incorporation of Al^3+^ into the hexaferrite structure (Figure 1d).

XRD patterns (Figure 1e) of both Ba_4_Co_2_Fe_36_O_60_ (*x* = 0.0) and Ba_4_Co_2_Fe_35_AlO_60_ (*x* = 1.0) samples displayed the characteristic reflections of the U-type hexaferrite structure, including the intense peak centered at approximately 2θ = 30.4°, confirming the successful formation of the desired crystalline phase. No secondary crystalline phases were detected. The average crystallite sizes, estimated using the Scherrer equation, were approximately 40.5 nm and 49.4 nm for the *x* = 0.0 and *x* = 1.0 samples, respectively.

Figure 1f presents the magnetic hysteresis (M–H) loops of the selected U-type hexaferrite particles (*x* = 0.0 and *x* = 1.0). Both compositions exhibited well-defined ferrimagnetic behavior, characterized by narrow hysteresis loops with low coercivity and remanent magnetization, indicating a soft magnetic nature. The Al^3+^-substituted ferrite displayed a slightly lower saturation magnetization (M_s_ = 44.5 Am^2^/kg) than the non-substituted composition (M_s_ = 47.0 Am^2^/kg), which can be attributed to the partial replacement of magnetic Fe^3+^ ions by non-magnetic Al^3+^ ions within the crystal lattice. Nevertheless, both particles retained high magnetic responsiveness, demonstrating that Al^3+^ substitution did not compromise their magnetic functionality. Furthermore, Al^3+^ non-substituted and substituted particles showed a coercivity (H_c_) value of 5.3 kAm^−1^, indicating that they are all magnetically soft. These characteristics make both compositions suitable for the fabrication of magnetically responsive hydrogel scaffolds that can be remotely manipulated using external magnetic fields.

### 3.2. Synthesis and Characterization of Magnetic Inks

#### 3.2.1. Rheological Properties

Magnetoactive scaffolds have attracted considerable interest in biomedical applications because of their ability to enhance cell behavior and enable remote magnetic stimulation [36,37]. Based on the results previously reported for Al^3+^-substituted U-type hexaferrites, Ba_4_Co_2_Fe_36_−*_x_*Al*_x_*O_60_ [6], in terms of their structural, magnetic, electrical, and dielectric properties, the compositions containing the lowest (*x* = 0.0) and highest (*x* = 1.0) Al^3+^ contents were selected for this study. In addition, two different particle loadings were evaluated to investigate the effect of particle concentration on the properties of the resulting inks and 3D-printed scaffolds. Thus, 20 or 200 mg of Ba_4_Co_2_Fe_36_−*_x_*Al*_x_*O_60_ (*x* = 0.0 or 1.0) were incorporated into 10 mL of a Gel–Alg formulation to prepare magnetic inks. The formulations were designated as GA (Gel-Alg control), GA-x0-20 (Gel-Alg containing 0.2 wt% Ba_4_Co_2_Fe_36_−*_x_*Al*_x_*O_60_, *x* = 0.0), GA-x0-200 (Gel-Alg + 2 wt% Ba_4_Co_2_Fe_36_−*_x_*Al*_x_*O_60_, *x* = 0.0), GA-x1-20 (Gel-Alg + 0.2 wt% Ba_4_Co_2_Fe_36_−*_x_*Al*_x_*O_60_, *x* = 1.0), and GA-x1-200 (Gel-Alg + 2 wt% Ba_4_Co_2_Fe_36_−*_x_*Al*_x_*O_60_, *x* = 1.0). To investigate whether both the composition and the loading of the magnetic phase influence the printability of the inks, rheological analyses were performed on Gel-Alg formulations.

**Figure 2:**
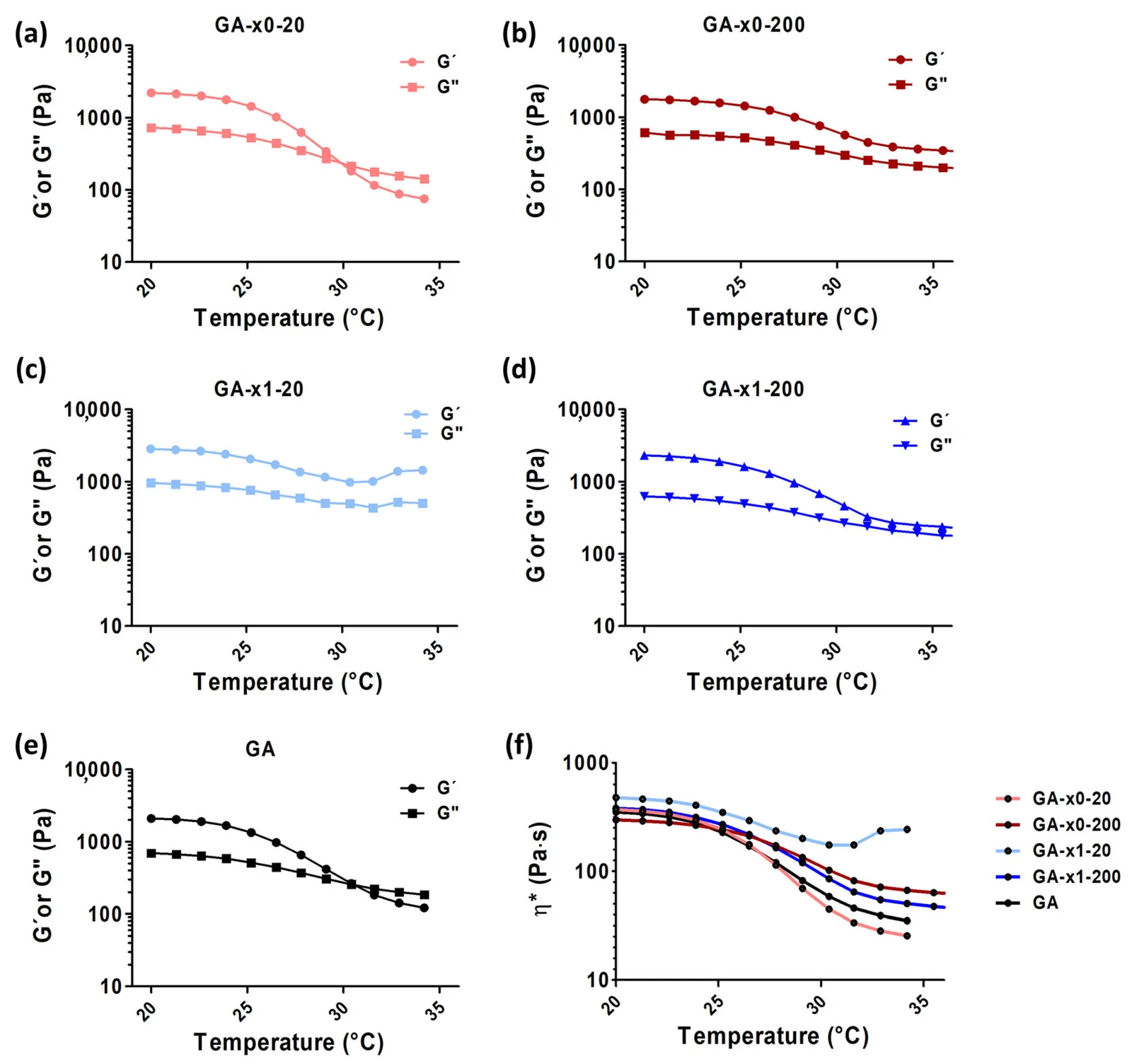
Rheological characterization of GA (black), GA-x0-20 (pink), GA-x0-200 (red), GA-x1-20 (light blue) and GA-x1-200 (blue). (**a**–**e**) Temperature-dependent evolution of the storage modulus (G′, circles) and loss modulus (G″, squares). (**f**) Temperature dependence of the complex viscosity (η*) for all formulations.

Since viscoelastic properties of gelatin are strongly affected by temperature [38], increasing the printing temperature is a common strategy in extrusion-based printing to reduce the viscosity of Gel-containing inks, thereby improving their flow through the printing nozzle [39,40]. Therefore, understanding the temperature-dependent rheological behavior of the developed formulations was essential for optimizing both printability and shape fidelity. These measurements provided valuable information on the sol–gel transition of the HMP-containing inks (Figure 2). At the initial temperature (20 °C), all formulations displayed a G′ value higher than the G″ one, indicating a predominantly elastic behavior before heating (Supplementary Material, Figure S1) [41]. As expected for physically crosslinked Gel-Alg hydrogels, the inks exhibited a progressive decrease in both modulus (Figure 2a–e), as well as in the complex viscosity (η*) (Figure 2f), as the temperature increased from 20 to 35.5 °C. This behavior reflects the gradual thermal disruption of the physically crosslinked polymer network upon heating. However, the magnitude of this reduction depended on both the concentration and composition of the incorporated hexaferrite particles. The GA-x0-20 formulation (Figure 2a) and control GA hydrogel (Figure 2e) exhibited a pronounced decrease in G′, which converged with G″ at around 30 °C, indicating a substantial loss of elastic behavior as the hydrogel approached the sol–gel transition. This suggests that the incorporation of a low concentration of non-substituted hexaferrites was insufficient to significantly reinforce the polymeric network. In contrast, increasing the particle loading to 200 mg clearly improved the thermal resistance of the gel network. Both GA-x0-200 (Figure 2b) and GA-x1-200 (Figure 2d) exhibited higher G′ values throughout the temperature range, with G′ exceeding G″, indicating the preservation of the elastic behavior.

These results indicate that increasing the HMP content effectively reinforces the Gel-Alg network and delays the thermal softening of the hydrogel. Interestingly, the GA-x1-20 formulation exhibited a distinct thermal response compared with the other inks (Figure 2c). Although G′, G″ and η* initially decreased with increasing temperature, G′ and η* showed a partial recovery around 32 °C. While the mechanism responsible for this phenomenon remains under study, it may indicate a temperature-induced rearrangement of the polymer–particle network promoted by the Al^3+^-substituted U-type hexaferrite particles, leading to enhanced structural stability during printing extrusion.

**Figure 3:**
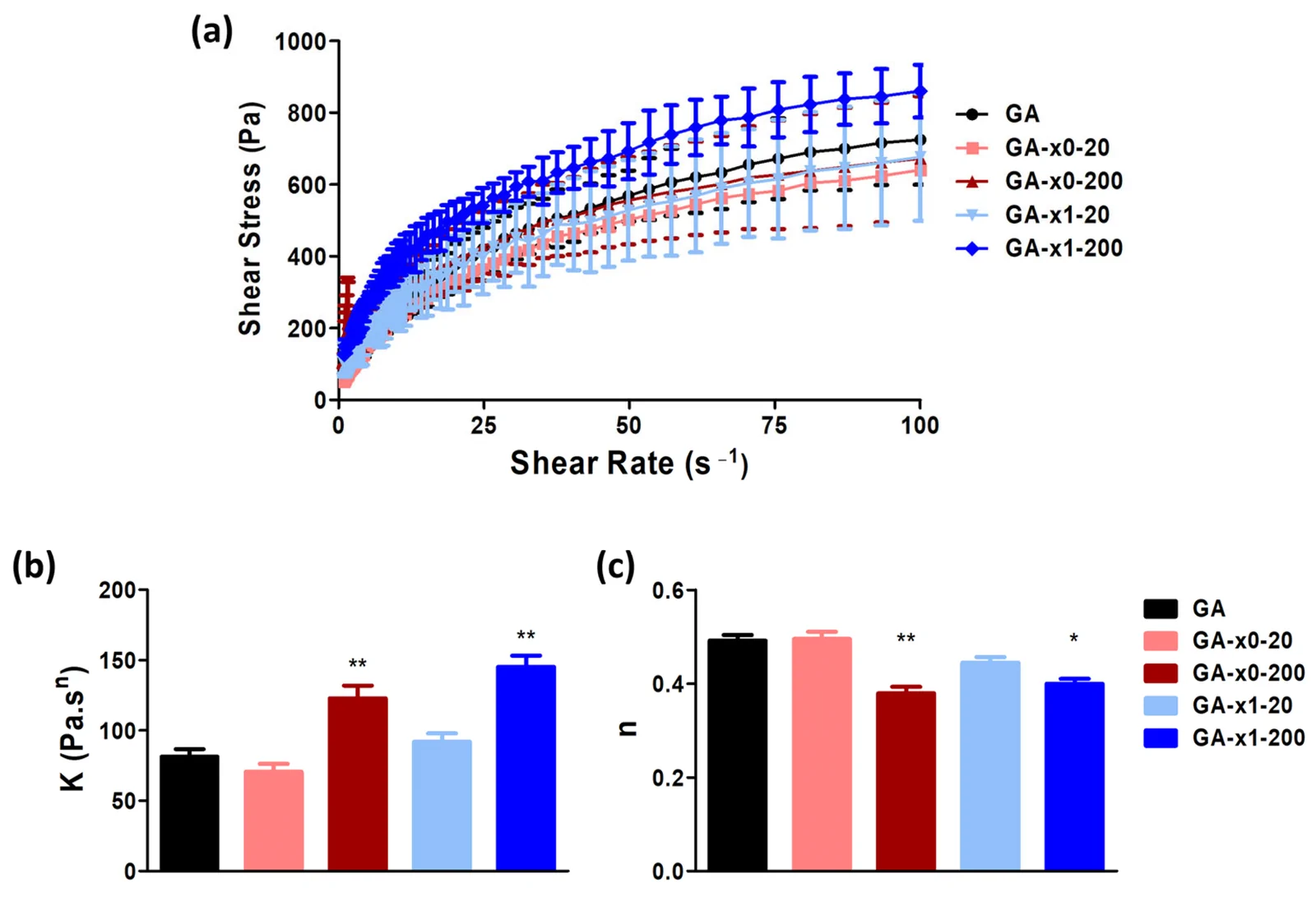
Rheological flow behavior of the Gel-Alg inks at 35.5 °C. (**a**) Shear stress as a function of shear rate for GA (black), GA-x0-20 (pink), GA-x0-200 (red), GA-x1-20 (light blue), and GA-x1-200 (blue). Experimental data were fitted using the Power Law model. (**b**) Consistency index (K) and (**c**) flow behavior index (n) calculated from the fitted curves. Values are expressed as mean ± SD (n = 2). Statistical significance is indicated as follows: * *p* < 0.05 and ** *p* < 0.01 compared with the GA control.

Figure 2 shows that some Gel-containing inks still exhibited a predominantly elastic gel-like response at 30 °C. Therefore, 35.5 °C was selected as a common temperature for the flow measurements to reduce the contribution of the elastic gel structure across all formulations and enable a more consistent comparison of their flow behavior under shear. This temperature was selected only for rheological characterization. As a result, all formulations exhibited a typical non-Newtonian shear-thinning behavior, characterized by a progressive decrease in apparent viscosity with increasing shear rate (Figure 3a). Such behavior is highly desirable for extrusion-based 3D printing because it facilitates the flow of the inks through the printing nozzle. To quantitatively compare the different formulations, the experimental data were fitted using the Power Law model (Equation (2)) [33], yielding high coefficients of determination (R^2^ > 0.98) for all samples. The calculated consistency index (K) and flow behavior index (n) are presented in Figure 3b and Figure 3c, respectively. In agreement with the temperature-dependent rheological behavior discussed above, K was also strongly influenced by particle loading. Both formulations containing 200 mg of HMP (GA-x0-200 and GA-x1-200) exhibited significantly higher K values than the control GA hydrogel, indicating greater resistance to flow and suggesting that increasing particle concentration reinforces the polymeric network. In contrast, formulations containing 20 mg of particles showed K values closer to that of the control, indicating a less pronounced reinforcing effect. The flow behavior index remained below 0.5 for all formulations, confirming their pronounced pseudoplastic character [42]. Moreover, increasing the particle loading resulted in lower n values, indicating a stronger shear-thinning response.

Overall, the rheological characterization demonstrates that all gel-containing ink formulations exhibited favorable rheological features for extrusion-based 3D printing, including a stable gel-like behavior (G′ > G″) and pronounced shear-thinning properties. More importantly, the results reveal that not only the concentration of the magnetic particles but also their chemical composition significantly influence the rheological performance of the inks. While increasing HMP loading reinforced the polymeric network by increasing the consistency index and enhancing the shear-thinning behavior, Al^3+^ substitution further modified the elastic response of the hydrogels. Therefore, both particle composition and concentration constitute complementary design parameters for engineering magnetically responsive inks with tunable rheological properties.

**Figure 4:**
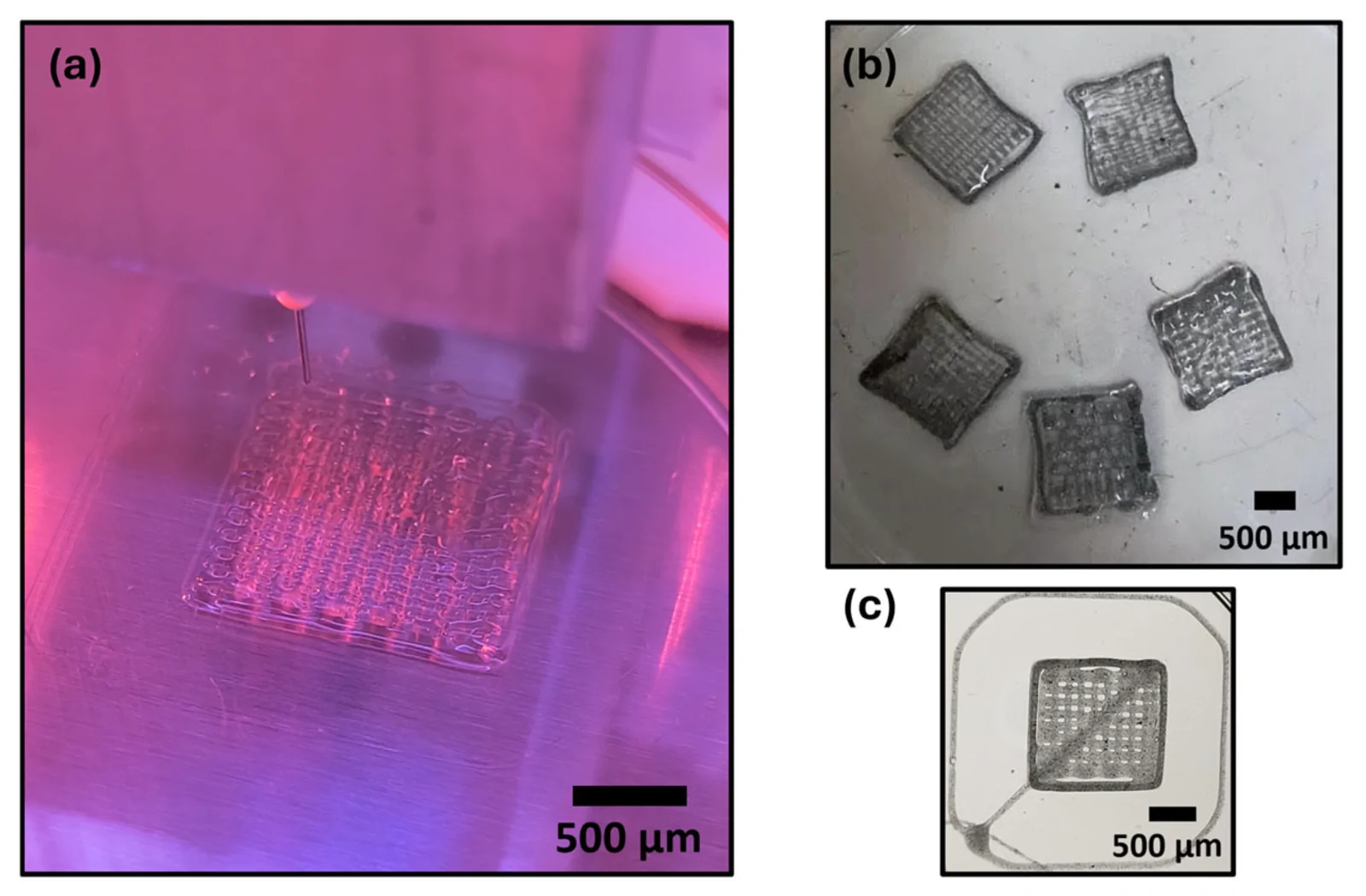
(**a**) 3D-printing of magnetic scaffolds by extrusion using a Life SI 3D bioprinter. (**b**,**c**) Representative scaffolds obtained by 3D-printing using the GA-x1-200 ink.

Considering this rheological characterization, printing was performed at 30 °C, where the inks exhibited an optimal balance between reduced viscosity, facilitating smooth extrusion through the printing nozzle, and sufficient elastic behavior (G′ > G″) to preserve filament shape after deposition. Under these conditions, the inks were successfully processed by extrusion-based 3D printing to fabricate square-grid scaffolds (15 × 15 × 2 mm), as shown in Figure 4.

#### 3.2.2. Printability Test

Printability can be considered a key property of biomaterials intended for 3D printing, reflecting their ability to be processed into structures with accurate and reproducible geometries while preserving the functional characteristics of the material. Therefore, printability involves aspects such as shape accuracy, shape fidelity, and material functionality [43]. The regularity and definition of the grid holes can be used to assess the geometric accuracy and shape fidelity of the printed constructs. In particular, the formation of regular square-grid holes with well-defined edges and corners is indicative of good printing performance and high shape fidelity.

Pr values were calculated for each magnetic ink after 3D printing of the square-grid scaffolds. Application of Equation (3) yielded Pr values of 1.07 ± 0.34 for GA, 1.04 ± 0.17 for GA-x0-20, 0.94 ± 0.31 for GA-x0-200, 0.91 ± 0.24 for GA-x1-20, and 0.95 ± 0.29 for GA-x1-200, with no statistically significant differences among the formulations (one-way ANOVA, *p* > 0.05). According to previously reported criteria, Pr values between 0.9 and 1.1 indicate acceptable printability, with Pr = 1 corresponding to an ideal square pore geometry [44,45]. These results indicate that the developed magnetic inks maintained acceptable printability and geometric fidelity under the conditions investigated, regardless of the hexaferrite composition or particle loading.

### 3.3. Characterization of Magnetic Scaffolds

#### 3.3.1. FE-SEM and EDS Analysis

**Figure 5:**
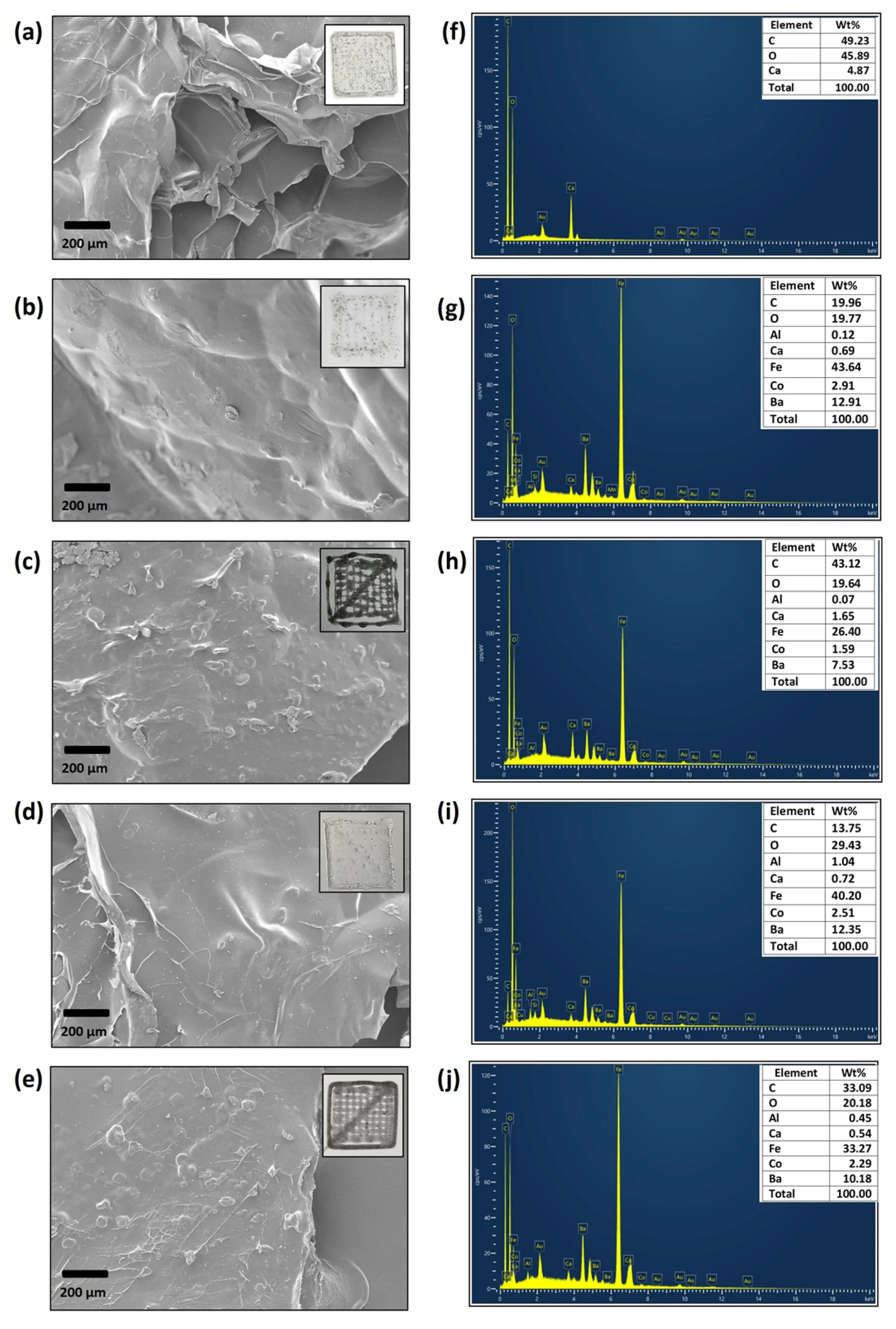
FE-SEM images (**a**–**e**) and EDS analysis (**f**–**j**) of 3D-printed scaffolds. (**a**,**f**) GA control; (**b**,**g**) GA-x0-20; (**c**,**h**) GA-x0-200; (**d**,**i**) GA-x1-20; (**e**,**j**) GA-x-200.

After printing, magnetic scaffolds were analyzed by FE-SEM. As shown in Figure 5a, the cross-sectional FE-SEM image of the control scaffold revealed a porous lamellar microstructure with an average pore size of 162 ± 21 µm. Such an interconnected porous structure is a common feature of hydrogel-based scaffolds and plays an important role in their biological performance for connective tissue formation. The porous architecture facilitates the transport of nutrients and oxygen, waste removal, cell infiltration, and extracellular matrix deposition [46]. Pore sizes within this range have also been reported to provide a suitable microenvironment for attachment and proliferation of skin fibroblasts, highlighting the potential of the developed scaffolds for tissue engineering applications [47,48,49,50]. Extrusion-based 3D printing represents a versatile and effective approach for producing hydrogel scaffolds with this well-defined and reproducible architecture. Unlike conventional fabrication techniques, this method enables precise control over scaffold geometry, pore organization, and filament spacing, allowing the design of constructs tailored to specific tissue engineering applications [51]. Such control is particularly advantageous for wound healing, where scaffold architecture strongly influences both the mechanical properties and the biological response.

Furthermore, scaffolds containing higher hexaferrite loadings exhibited a greater number of incorporated particles (Figure 5c,e). Although individual particle morphology could not be clearly distinguished, as they were embedded within the GA matrix, EDS analysis (Figure 5g–j) unequivocally confirmed their successful incorporation into the scaffolds. The characteristic signals corresponding to Fe, Ba, Co, O and Al (for GA-x1-20 and GA-x1-200) detected in the magnetic scaffolds are consistent with the elemental composition of the U-type hexaferrites, whereas these elements were absent in the control material. As expected, the EDS spectrum of the control scaffold (Figure 5f) exhibited only the characteristic signals of C and O, arising from the biopolymer matrix, together with Ca, which originated from the CaCl_2_ crosslinking process.

The homogeneous incorporation of the ferrite particles into the hydrogel network is particularly important, as a uniform particle distribution is expected to promote a more homogeneous magnetic response throughout the scaffold while minimizing the formation of particle-rich aggregates that could compromise the structural integrity or biological performance of the constructs. Moreover, the encapsulation of the particles within the polymeric matrix may reduce the particle release during handling or implantation, contributing to the stability of the composite scaffold [52].

#### 3.3.2. FTIR Analysis

**Figure 6:**
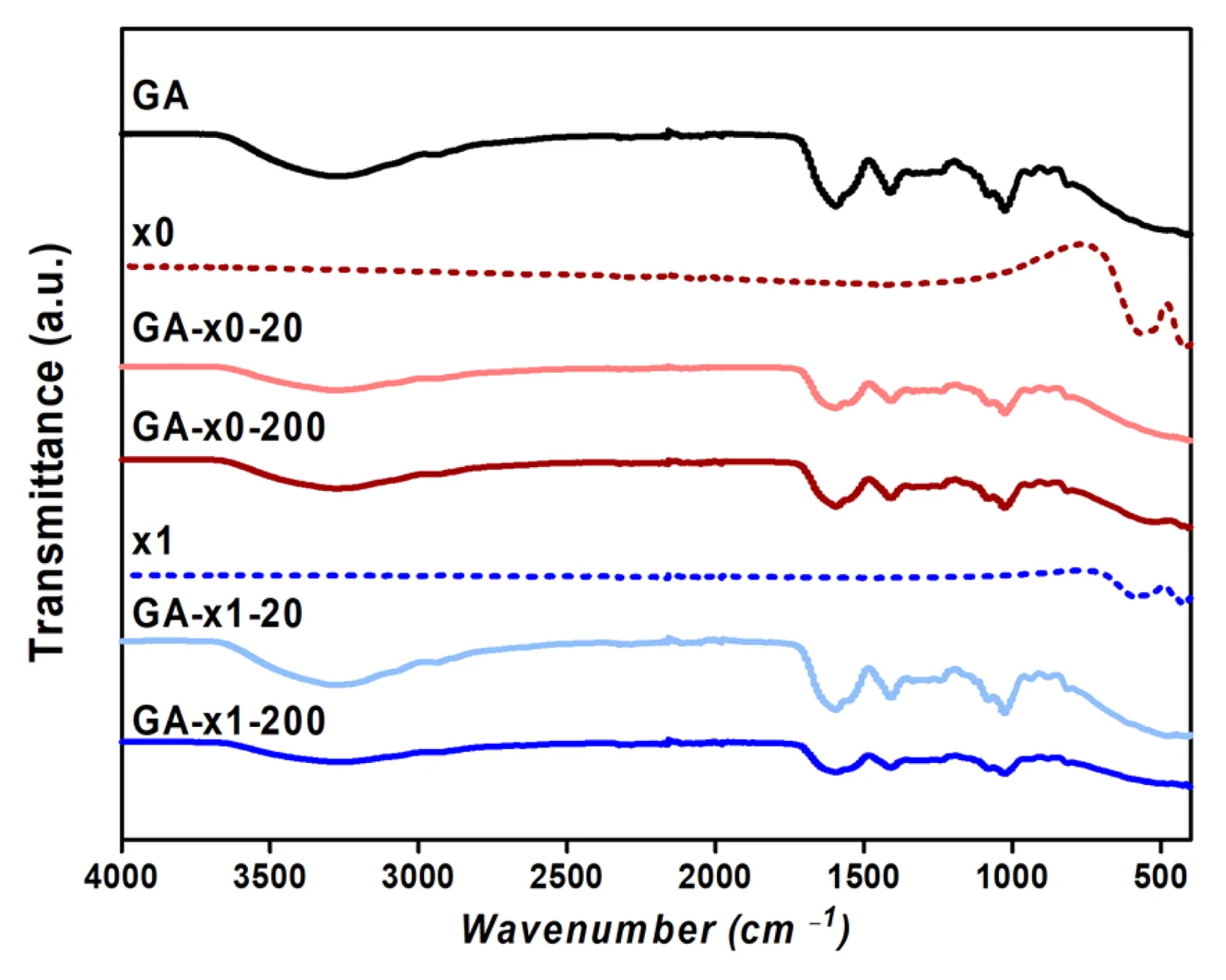
FTIR spectra of GA (black), HMP (x0: red striped line; x1: blue striped line) and magnetic GA scaffolds (GA-x0-20: pink, GA-x0-200: red, GA-x1-20: light blue, GA-x1-200: blue).

Figure 6 presents the FTIR spectra of the HMP, the GA control scaffold, and the magnetic GA scaffolds. With the exception of the spectra corresponding to the hexaferrite particles, all samples exhibited the characteristic absorption bands of Gel and Alg, indicating that the incorporation of the magnetic particles did not alter the chemical structure of the polymeric matrix. In fact, these spectra reveal the successful formation of the polymeric network through intermolecular interactions between the two biopolymers [53]. The characteristic bands detected at 1079 and 1031 cm^−1^ are assigned to the C–O–C and C–O stretching vibrations of the guluronic and mannuronic units of Alg, respectively [54]. The absorption band at 1542 cm^−1^ is attributed to the amide II vibration of Gel, involving N–H bending and C–N stretching [55]. In addition, a broad absorption signal centered at 3272 cm^−1^ was assigned to the overlapping stretching vibrations of hydroxyl (O–H) and amide (N–H) groups of amides. The band observed at 1641 cm^−1^ corresponds to the overlap of the amide I vibration of Gel, mainly associated with C=O stretching, and the asymmetric stretching vibration of the carboxylate (COO^−^) groups of Alg [56]. Interestingly, the characteristic FTIR bands of the hexaferrite particles, observed in the 400–600 cm^−1^ region, were not clearly resolved in the spectra of the magnetic scaffolds, only slightly more evident at the highest ferrite loading (200 mg). This finding can be attributed to the lower concentration of the particles relative to the polymeric matrix, resulting in the predominance of the Gel and Alg absorption bands. Overall, the preservation of the characteristic band positions confirms that the incorporation of the hexaferrite particles is governed by physical entrapment and non-covalent interactions (such as hydrogen bonding) within the polymeric network rather than chemical modifications.

#### 3.3.3. Swelling Capacity

The swelling behavior of hydrogels is a critical property for wound healing applications, as it determines their ability to absorb wound exudates, facilitate the transport of nutrients and metabolic waste, and regulate the diffusion and release of therapeutic agents [57]. Consequently, highly swellable hydrogels have attracted considerable attention for wound dressings and tissue engineering scaffolds [58].

**Figure 7:**
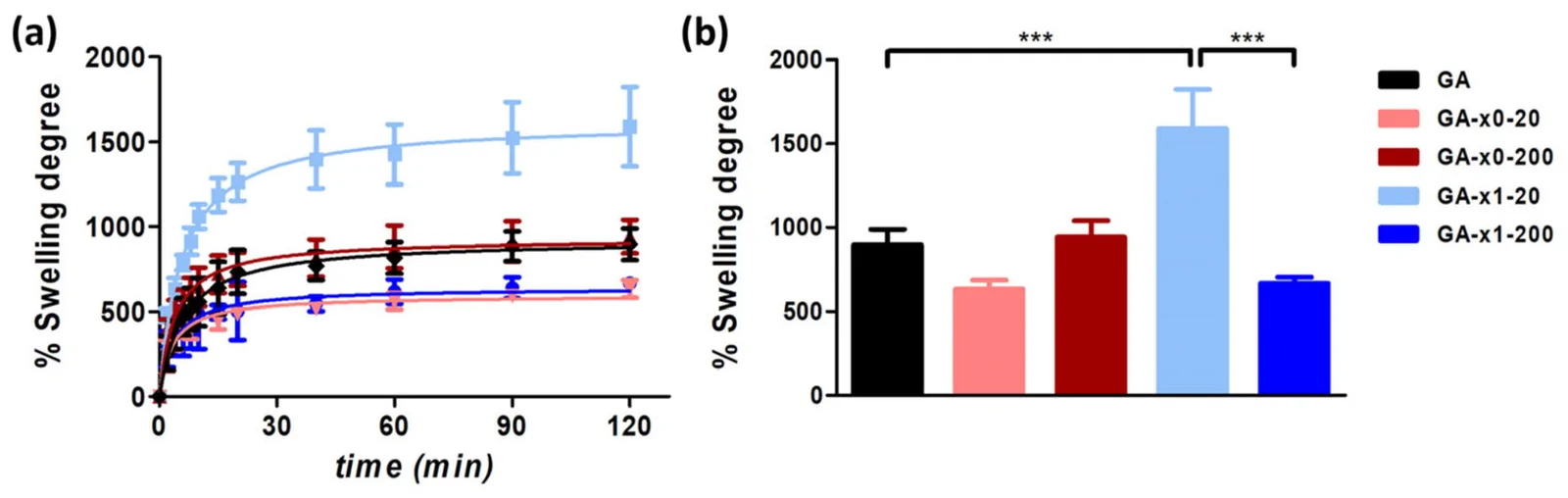
Swelling behavior of the 3D-printed hydrogels. (**a**) Swelling degree (%) of GA (black), GA-x0-20 (pink), GA-x0-200 (red), GA-x1-20 (light blue), and GA-x1-200 (blue) as a function of immersion time in distilled water. (**b**) Equilibrium swelling degree after 120 min of hydration. Data are presented as mean ± SD (n = 3). Statistical significance was determined by one-way ANOVA followed by Tukey’s post hoc test (*** *p* < 0.001).

Figure 7 shows the swelling capacity of magnetic scaffolds. All formulations exhibited a rapid water uptake during the first minutes of immersion, followed by a plateau after approximately 20–30 min, indicating that the hydrogel network reached swelling equilibrium within a relatively short period. This swelling behavior is consistent with the porous lamellar architecture observed by FE-SEM, as the interconnected pore network facilitates rapid water diffusion into the polymeric matrix until swelling equilibrium is reached [59]. Furthermore, it was found that the swelling behavior depended on the Al^3+^ substitution level, indicating that water uptake was governed by the interaction between the particles and the polymeric network. Interestingly, the GA-x1-20 scaffold exhibited the highest swelling ratio among all formulations (1590 ± 235%). This behavior is consistent with its distinctive rheological response (Figure 2), suggesting that the incorporation of a low concentration of Al^3+^-substituted hexaferrite particles modifies the organization of the Gel-Alg polymeric network. In addition, the enhanced swelling may be related to the presence of Al^3+^ in the substituted hexaferrite, since Al^3+^-containing materials have been reported to exhibit a high affinity for water owing to the increased surface hydroxylation and hydrophilicity of the oxide surface [60]. Together, these findings suggest that, at low particle loadings, Al^3+^ substitution may promote the formation of a material capable of absorbing larger amounts of water while preserving sufficient structural integrity. However, increasing the content of the same Al^3+^-substituted ferrite to 200 mg resulted in a marked reduction in the swelling ratio, yielding values comparable to those of the control GA scaffold. This finding suggests that, at higher particle concentrations, the reinforcing effect of the inorganic phase becomes dominant, restricting the expansion of the GA network during hydration, and ultimately limiting the swelling capacity.

### 3.4. Results and Discussion of the Functional Characterization of 3D-Printed Magnetic Scaffolds

#### 3.4.1. Magnetic Response

**Figure 8:**
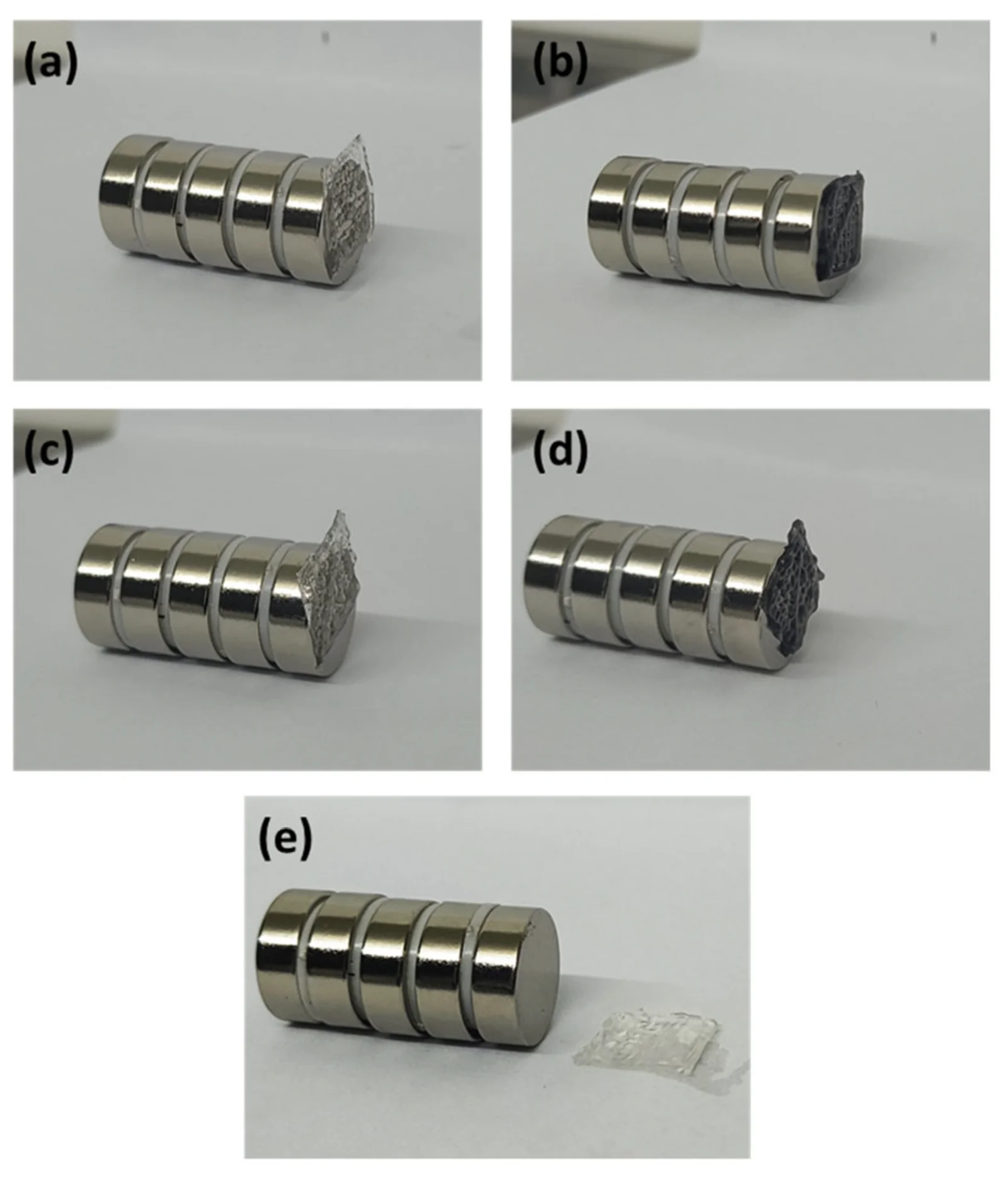
Magnetic response for 3D-printed scaffolds upon exposure to NdFeB magnets. (**a**) GA-x0-20, (**b**) GA-x0-200, (**c**) GA-x1-20, (**d**) GA-x1-200, and (**e**) control GA.

The magnetic responsiveness of the 3D-printed scaffolds was qualitatively evaluated by exposing the samples to an external permanent magnet. As shown in Figure 8 and Supplementary Videos S1–S4, all magnetic scaffolds exhibited a clear response toward the magnetic field, whereas the GA control remained unaffected (Supplementary Video S5). These observations confirm that the HMP retained their magnetic functionality after incorporation into the GA matrix and throughout the extrusion-based 3D printing and crosslinking processes. Moreover, the magnetic response was observed for all ferrite-containing formulations, regardless of the Al^3+^ substitution level or particle loading, demonstrating the successful fabrication of 3D-printed magnetically responsive hydrogel scaffolds. The ability of both ferrite compositions and both concentrations to impart magnetic responsiveness provides greater flexibility for tailoring scaffold formulations according to the desired properties while preserving their magnetic functionality. These findings highlight the potential of the developed scaffolds for further investigation as magnetically responsive platforms for tissue engineering, where external magnetic fields may be exploited to remotely modulate scaffold performance and cellular behavior. Future studies will include quantitative magnetic characterization of the developed materials to determine their magnetic properties more comprehensively and complement the qualitative response to an external magnet reported in the present work.

#### 3.4.2. Immune Cells Response

**Figure 9:**
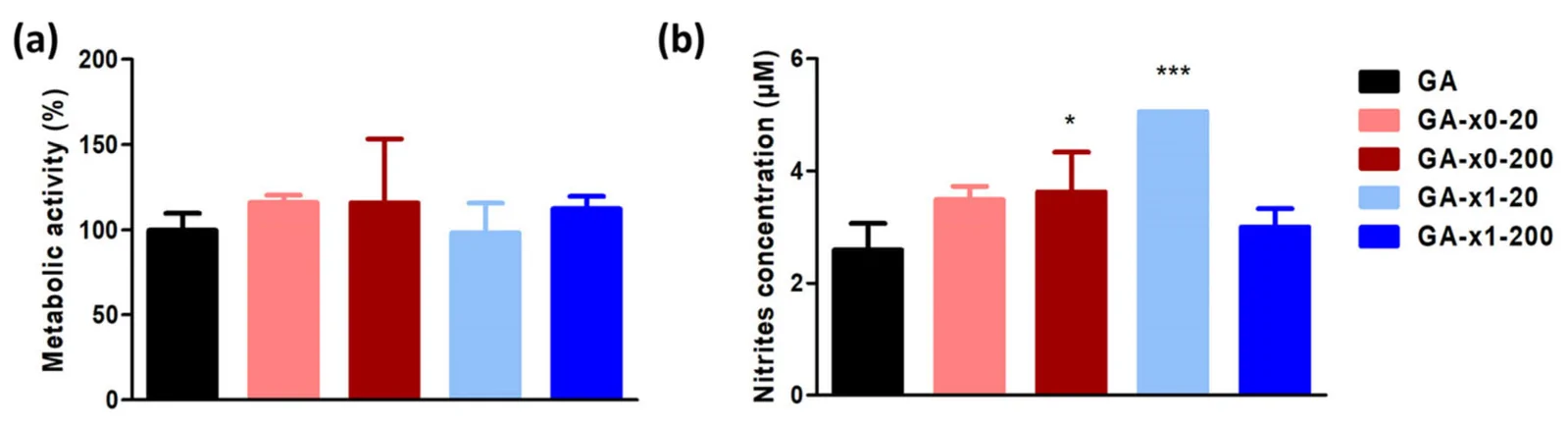
Macrophages response. (**a**) Metabolic activity (%) and (**b**) nitrite concentration (μM) after 24 h of incubating macrophages with GA (black) and magnetic GA scaffolds: GA-x0-20 (pink), GA-x0-200 (red), GA-x1-20 (light blue), and GA-x1-200 (blue). Data are presented as mean ± SD (n = 3). Statistical significance was determined by one-way ANOVA followed by Tukey’s post hoc test (*** *p* < 0.001, * *p* < 0.05).

Bioactive biomaterials are designed to create a favorable local microenvironment that supports tissue regeneration. However, following implantation, they may trigger an inflammatory response. Therefore, evaluating the response of immune cells, particularly macrophages, is essential to evaluate the biological performance of the new biomaterials. In the present study, RAW 264.7 macrophages were incubated with 3D-printed GA-based scaffolds to investigate their immunological response. Figure 9a shows that the incorporation of U-type hexaferrite particles into the Gel-Alg matrix did not significantly affect the metabolic activity of macrophages compared with the unloaded scaffold. These findings are further supported by our previous research that evidence that HMP exhibited good cytocompatibility toward fibroblasts [6].

Nitric oxide (NO) production is an indicator of macrophage activation toward a pro-inflammatory phenotype. Since NO is rapidly converted into nitrite in aqueous media, nitrite concentration was quantified using the Griess assay as an indirect measure of macrophage activation. The results (Figure 9b) further confirmed that the incorporation of HMP did not induce a pronounced inflammatory response. Although a statistically significant increase in nitrite production was detected for the GA-x1-20 formulation, the absolute nitrite concentration remained low (approximately 5 µM) and was considerably below the levels typically reported for LPS-activated RAW264.7 macrophages, which generally exceed 20 µM [61]. Therefore, these findings suggest that the observed increase in nitrite production may not reflect a pronounced pro-inflammatory response under the experimental conditions evaluated [35]. These results indicate that the magnetic scaffolds preserve, at short times, the favorable biological properties of the biopolymeric matrix while providing magnetic response, which is attractive for their use as stimuli-responsive platforms. In this context, further analysis in immune cells is necessary, evaluating metabolic activity and NO expression over longer time frames in addition to other parameters such as cytokine secretion and specific cell marker expression to evaluate scaffolds’ potential applications.

## 4. Conclusions

This work demonstrates the successful development of magnetically responsive Gel-Alg inks reinforced with U-type hexaferrite microparticles exhibiting different Al^3+^ substitution levels and particle loadings. Structural characterization confirmed the successful synthesis of the ferrite powders, while their incorporation into the hydrogel matrix did not compromise the extrusion-based 3D printing process, allowing the fabrication of 3D-printed scaffolds containing magnetic particles.

Rheological analyses revealed that both the concentration and chemical composition of the magnetic particles play a key role in tuning the viscoelastic behavior of the inks. Increasing the particle loading enhanced the consistency and thermal stability of the hydrogel network while preserving the pronounced shear-thinning behavior required for extrusion-based printing. In addition, Al^3+^-substituted ferrites induced distinct changes in the viscoelastic response, highlighting particle composition as an additional parameter for tailoring ink performance. All 3D-printed magnetic scaffolds retained the magnetic responsiveness of the incorporated particles, preserved macrophage metabolic activity, and did not induce a pro-inflammatory activation of macrophages.

Finally, although the U-type hexaferrites incorporate barium and cobalt, several structural and matrix factors mitigate potential toxicological concerns regarding heavy metal ion release and degradation. Unlike soluble metallic salts or less stable metal oxides, U-type hexaferrites possess a robust, tightly bound crystal framework that exhibits high chemical stability under physiological conditions, significantly restricting spontaneous ion leaching. Furthermore, embedding these ceramic particles within the ionically crosslinked gelatin–alginate matrix provides a secondary physical barrier, further impeding particle liberation and diffusion into the surrounding microenvironment. While the 24-h macrophage metabolic assay confirms short-term acute cytocompatibility, it serves as an initial screening step that does not fully capture chronic responses. Consequently, comprehensive long-term degradation assays, quantitative ion-release tracking via ICP-MS over extended culture periods, and multi-week viability studies remain essential next steps to fully establish the long-term safety profile of these magnetoactive scaffolds for future tissue-engineering applications.

Overall, these findings demonstrate that U-type hexaferrites constitute a versatile magnetic phase for engineering inks with tunable rheological and physicochemical properties. The possibility of independently modulating particle composition and loading provides a flexible strategy for designing magnetic scaffolds adapted to different tissue engineering applications. Future studies will focus on evaluating the biological performance of these 3D scaffolds under static and magnetically stimulated conditions to explore their potential for remotely controlled tissue regeneration.

## Data Availability

The data presented in this study are available on request from the corresponding author due to preserve the confidentiality of the results.

## Supplementary Materials

The following supporting information can be downloaded at: https://www.mdpi.com/article/10.3390/pharmaceutics18091186/s1. Figure S1: Characterizations of viscoelastic behavior of Gel-Alg inks: Frequency sweep tests at 20 °C of GA-x0-20 (a), GA-x0-200 (b), GA-x1-20 (c), GA-x1-200 GA (d) and GA (e). Storage modulus (G′, circles) and loss modulus (G″, squares). (f) Frequency dependence of the complex viscosity (η*) for all formulations. Temperature 20 °C. Videos: SV_S1_GA-x0-20; SV_S2_GA-x0-200; SV_S3_GA-x1-20; SV_S4_GA-x1-200; SV_S5_GA.
