# Supplementary material for: 3D-Printing of Magnetoactive Gelatin–Alginate Scaffolds
Source: Pharmaceutics. 2026 Sep 19;18(9):1186. doi: 10.3390/pharmaceutics18091186 (PMC13610972; doi:10.3390/pharmaceutics18091186)

3D-Printing of Magnetoactive Gelatin-Alginate Scaffolds

Sofia Municoy[1], Exequiel Giorgi[2,3], María Edith Farías[4], Romina B. Currá[4], Hina N. Chaudhari[5], Rajshree B. Jotania[5], Robert C. Pullar[6], Mauricio De Marzi[2,3,*], Martín F. Desimone[1]

Original Images

Figure 1a

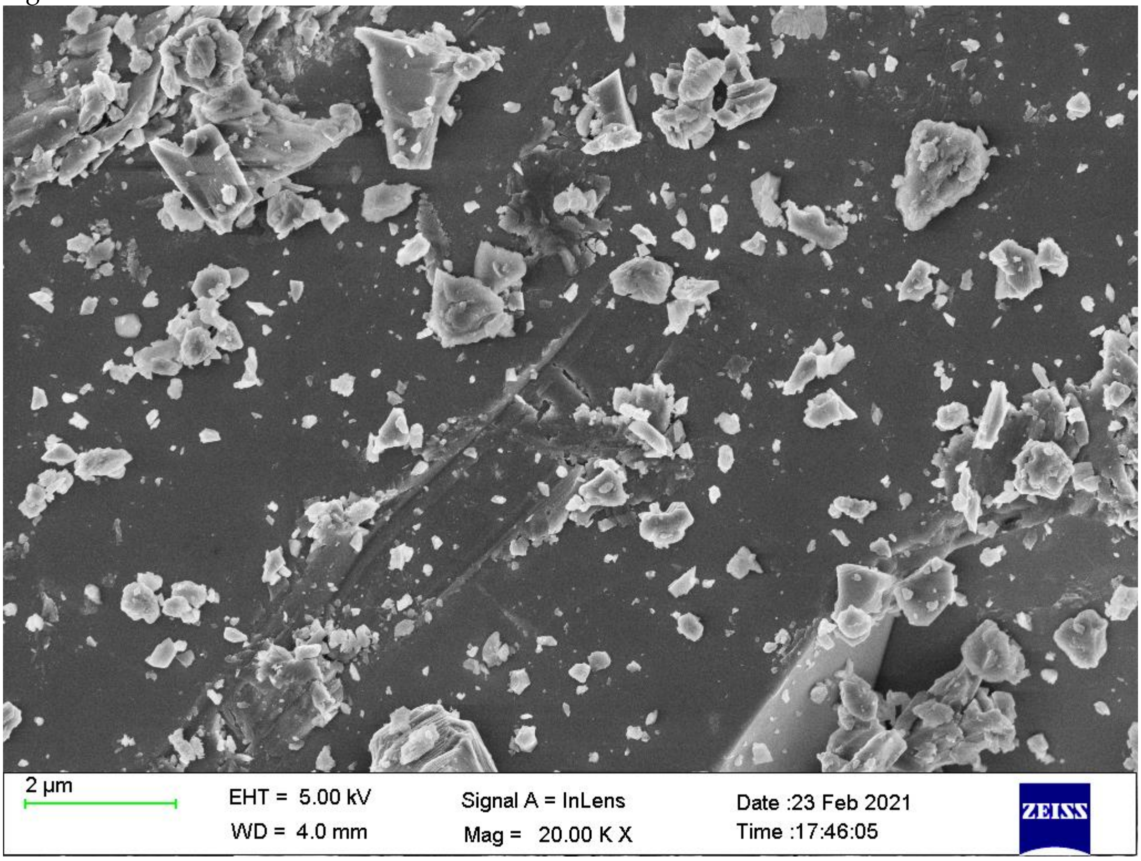

Figure 1c

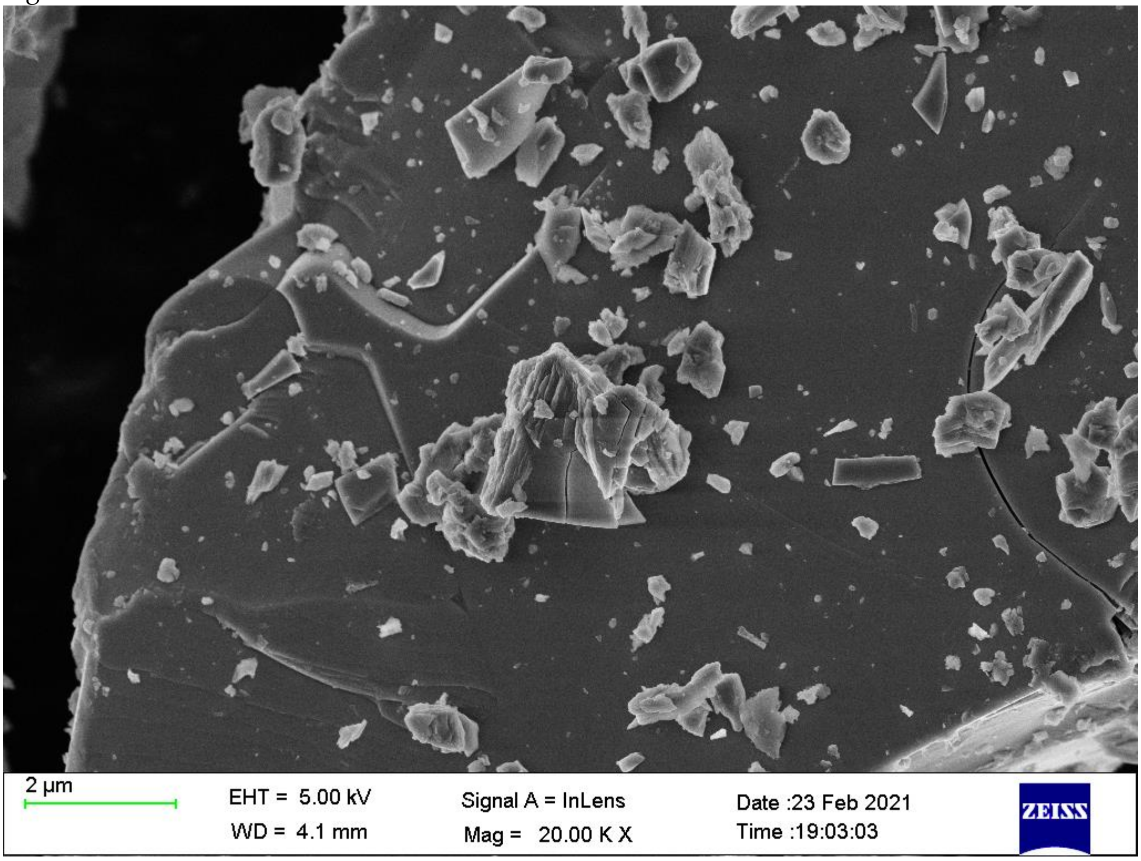

Figure 5a

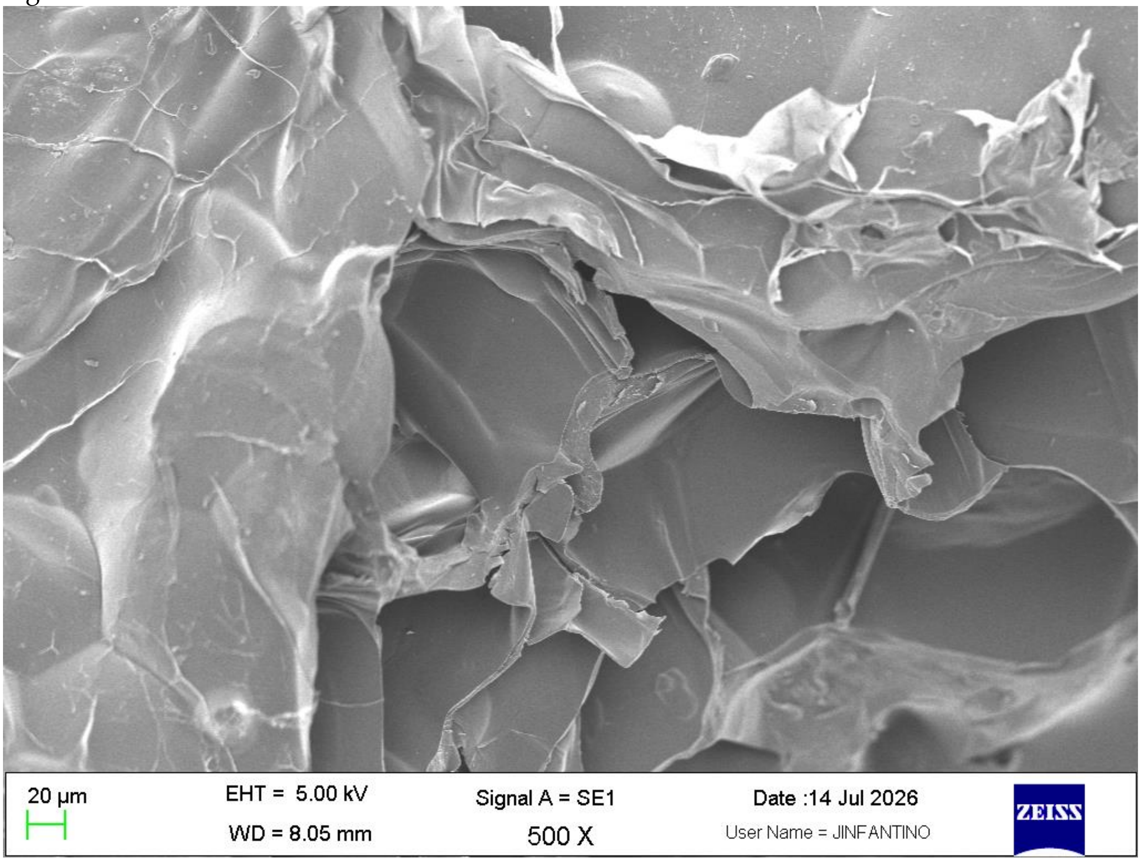

Figure 5b

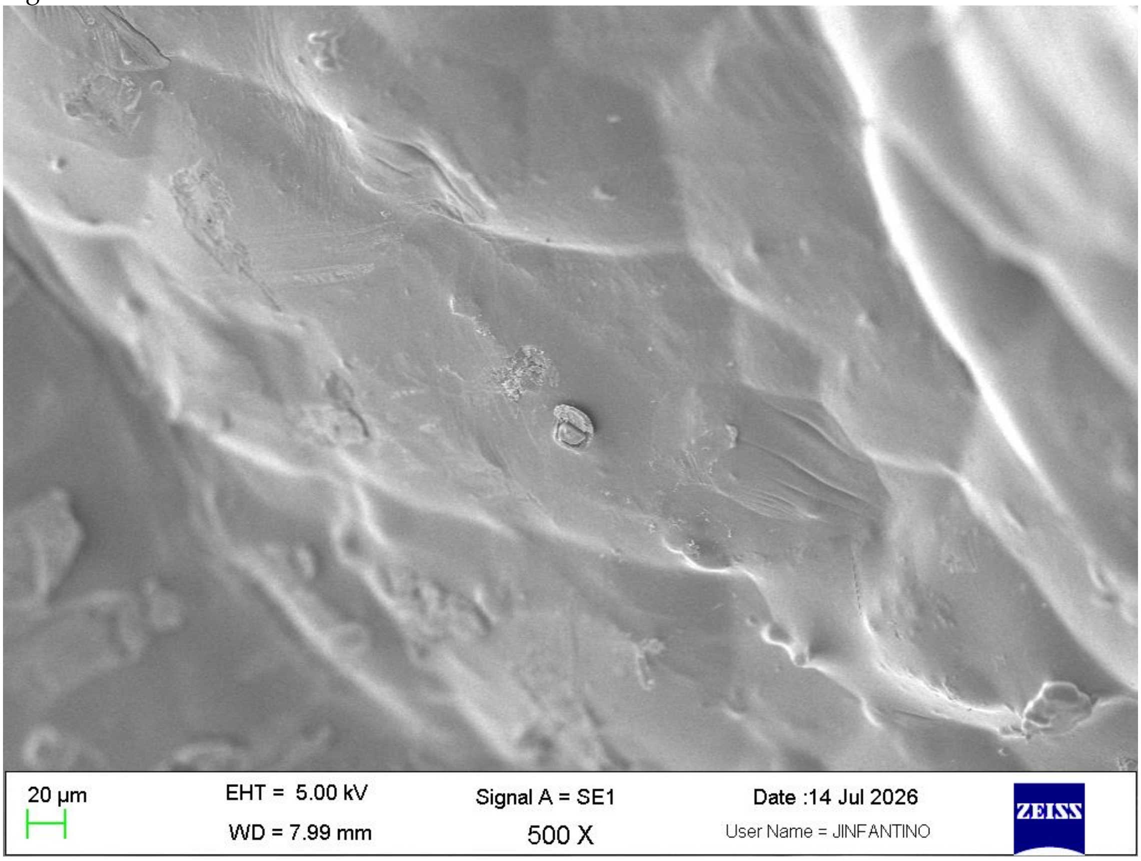

Figure 5c

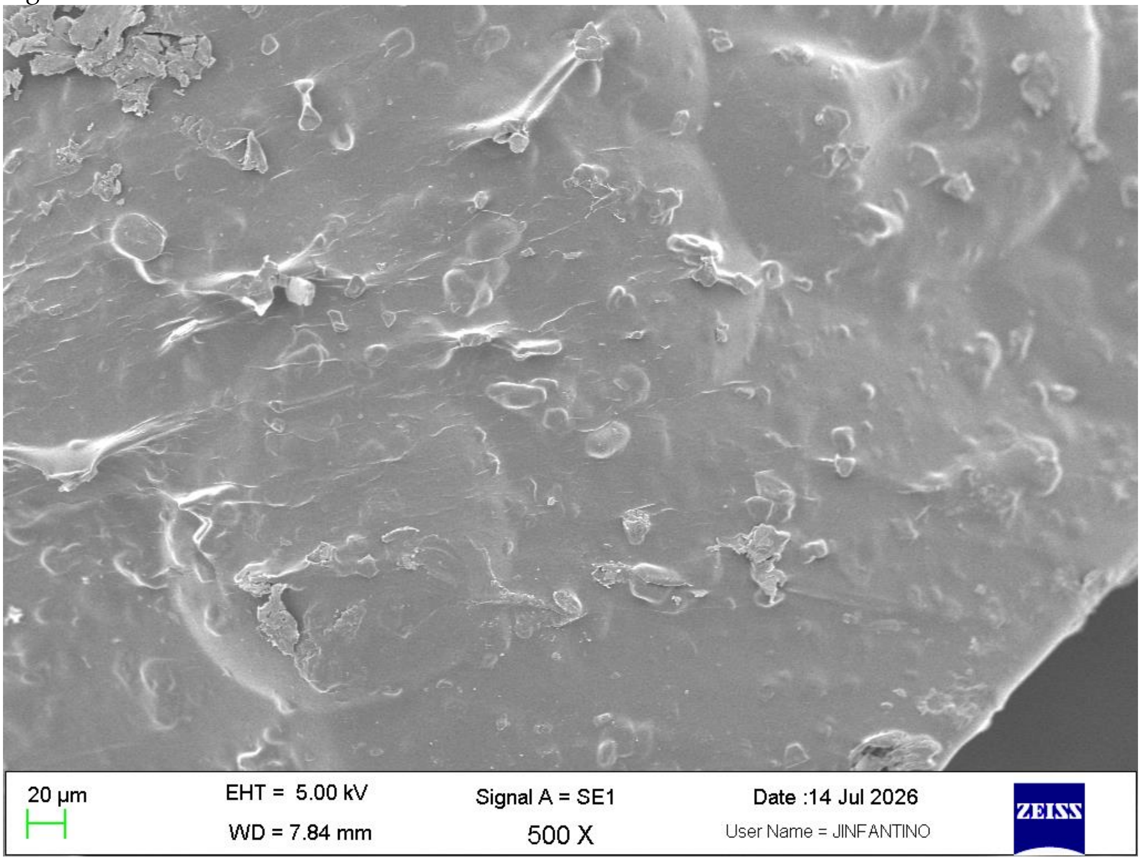

Figure 5d

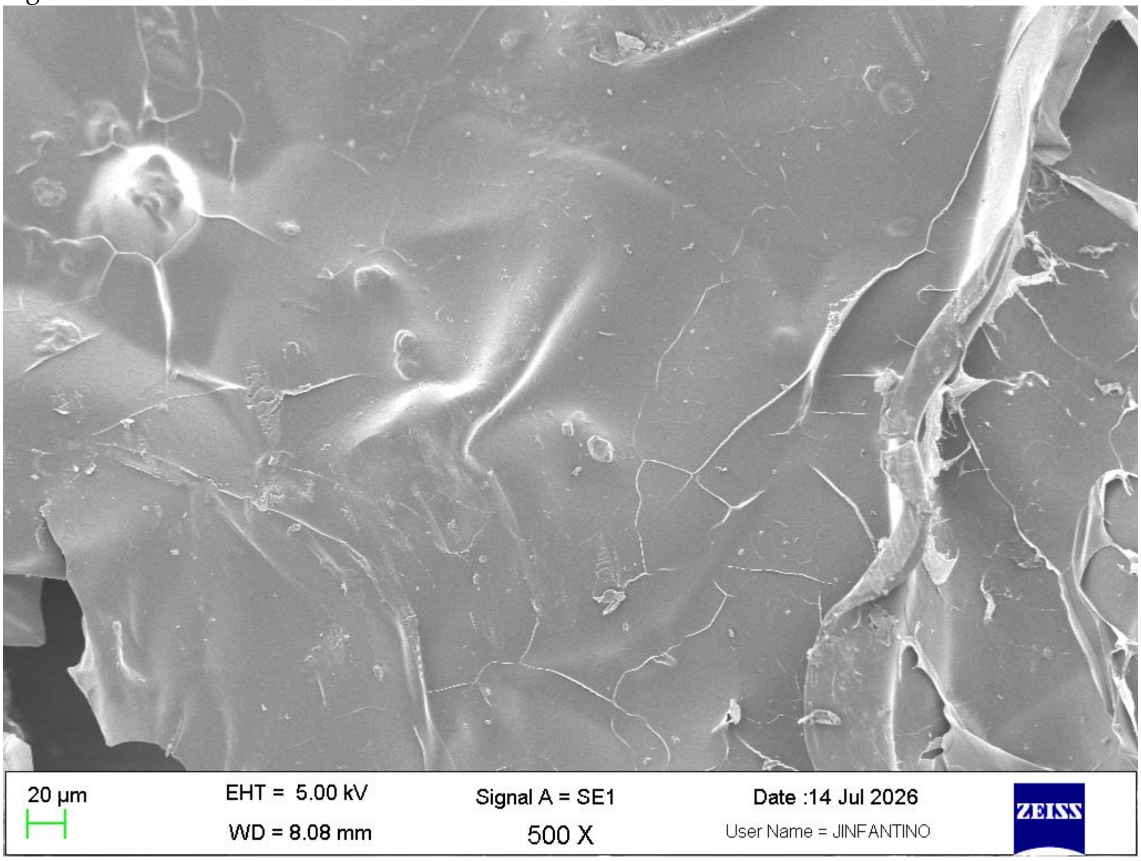

Figure 5e

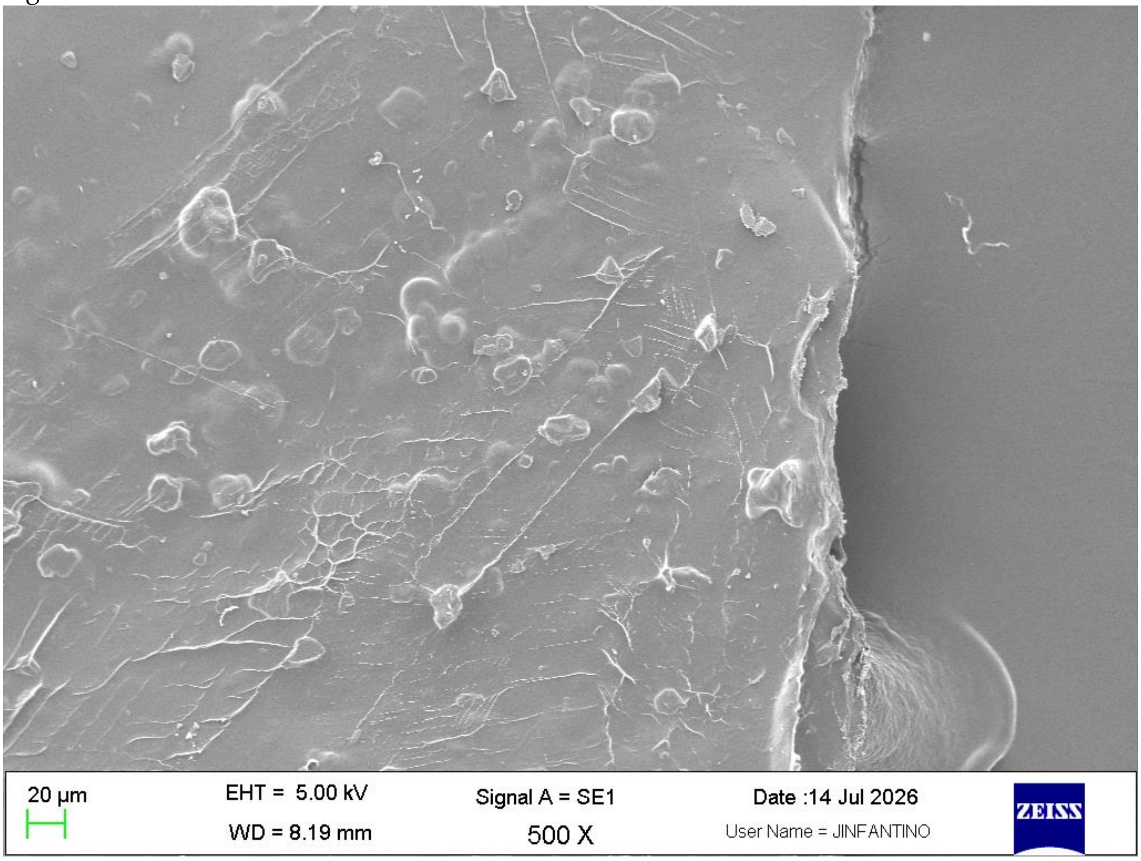

Supplement: Supplementary file 1 [file pharmaceutics-18-01186-s001.zip › pharmaceutics-4520149-S. Municoy et al Original Images.pdf]
