# Supplementary material for: 3D-Printing of Magnetoactive Gelatin–Alginate Scaffolds
Source: Pharmaceutics. 2026 Sep 19;18(9):1186. doi: 10.3390/pharmaceutics18091186 (PMC13610972; doi:10.3390/pharmaceutics18091186)
Supplement: Supplementary file 1 [file pharmaceutics-18-01186-s001.zip › pharmaceutics-4520149-supplementary material.pdf]

pharmaceutics

MDPI

Supplementary Material

3D-Printing of Magnetoactive Gelatin-Alginate Scaffolds

Sofia Municoy [1], Exequiel Giorgi [2,3], María Edith Farías [4], Romina B. Currá [4], Hina N. Chaudhari [5], Rajshree B. Jotania [5], Robert C. Pullar [6], Mauricio De Marzi [2,3,*] and Martín F. Desimone [1]

1 Universidad de Buenos Aires, Facultad de Farmacia y Bioquímica, Instituto de Química y Metabolismo del Fármaco (IQUIMEFA), Consejo Nacional de Investigaciones Científicas y Técnicas (CONICET), Junín 956, Buenos Aires 1113, Argentina; smunicoy@docente.ffyb.uba.ar (S.M.); desimone@ffyb.uba.ar (M.F.D.)
2 Grupo de Investigaciones Básicas y Aplicadas en Inmunología y Bioactivos (GIBAIB), Instituto de Ecología y Desarrollo Sustentable (INEDES), CONICET, Universidad Nacional de Luján, Av. Constitución y Ruta 5, Luján 6700, Buenos Aires, Argentina; egiorgi@unlu.edu.ar
3 Laboratorio de Inmunología, Departamento de Ciencias Básicas, Universidad Nacional de Lujan, Av. Constitución y Ruta 5, Luján, Buenos Aires, Argentina
4 Grupo de Investigación en Nanopartículas y Reología de Biopolímeros Alimenticios (GINaRBA), Departamento de Tecnología, Universidad Nacional de Luján, Av. Constitución y Ruta 5, Luján, Buenos Aires, Argentina; efarias@unlu.edu.ar (M.E.F.); rcurra@mail.unlu.edu.ar (R.B.C.)
5 Department of Physics, University School of Sciences, Gujarat University, Ahmedabad 380009, India; heenachaudhary0506@gmail.com (H.N.C.); rajshree_jotania@yahoo.co.in (R.B.J.)
6 Department of Molecular Science and Nanosystems (DSMN), Università Ca' Foscari, Venezia, 30172 Venice, Italy; robertcarlyle.pullar@unive.it
* Correspondence: mdemarzi@unlu.edu.ar

Viscoelastic behavior of inks

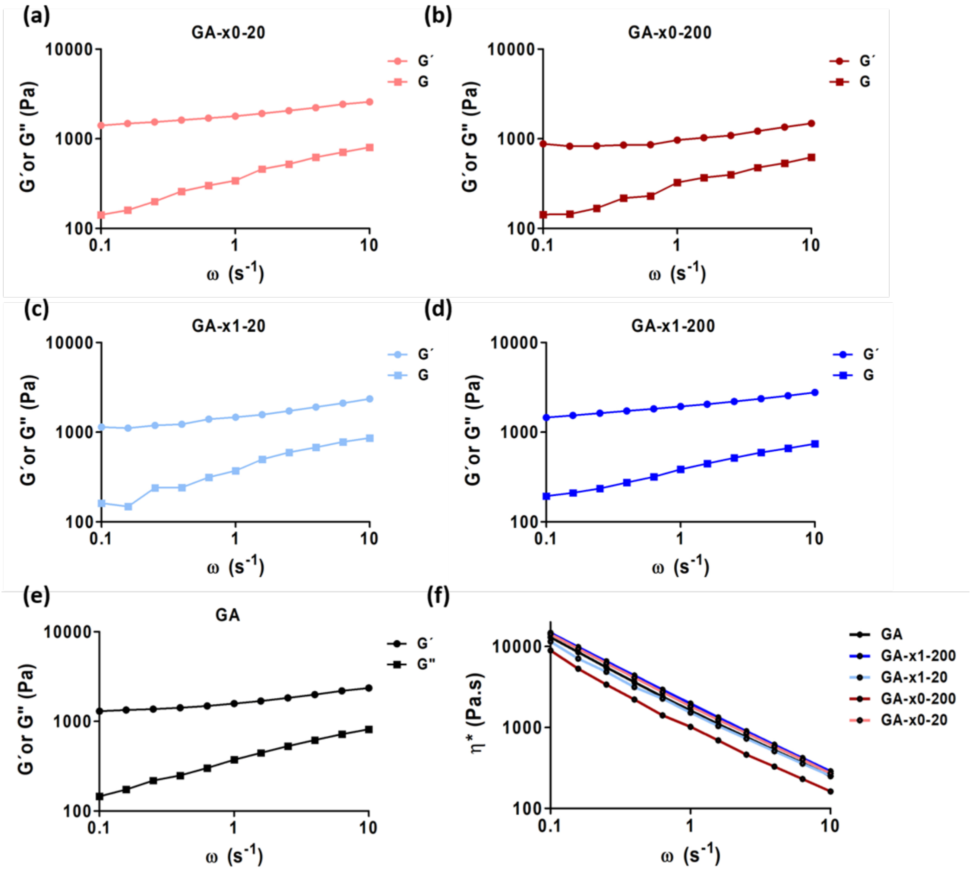

Figure S1. Characterizations of viscoelastic behavior of Gel-Alg inks: Frequency sweep tests at 20°C of GA-x0-20 (a), GA-x0-200 (b), GA-x1-20 (c), GA-x1-200 GA (d) and GA (e). Storage modulus (G′, circles) and loss modulus (G″, squares). (f) Frequency dependence of the complex viscosity (η*) for all formulations. Temperature 20 °C.

Figure S1(a-e) shows that in all Gel-Alg based inks, G′ exceeded G″ across the entire frequency range, indicating predominantly elastic, solid-like behavior. Complex viscosity (η*) is a measure of the material's resistance to flow during oscillatory deformation. The treatments do not significantly alter the η* (Fig. 1Sf). All formulations exhibited a progressive decrease in η* with increasing angular frequency, reflecting the disruption and alignment of the internal structure under oscillatory shear. The curves are nearly parallel, indicating that they share the same relaxation mechanism.

https://doi.org/10.3390/xxxxx
